# Effect of Electric Fields on the Decomposition of Phosphate Esters

**DOI:** 10.1021/acs.jpcc.4c04412

**Published:** 2024-09-12

**Authors:** Zhaoran Zhu, James P. Ewen, Efstratios M. Kritikos, Andrea Giusti, Daniele Dini

**Affiliations:** † Department of Mechanical Engineering, Imperial College London, London SW7 2AZ, U.K.; ‡ Department of Applied Physics and Materials Science, California Institute of Technology, Pasadena, California 91125, United States

## Abstract

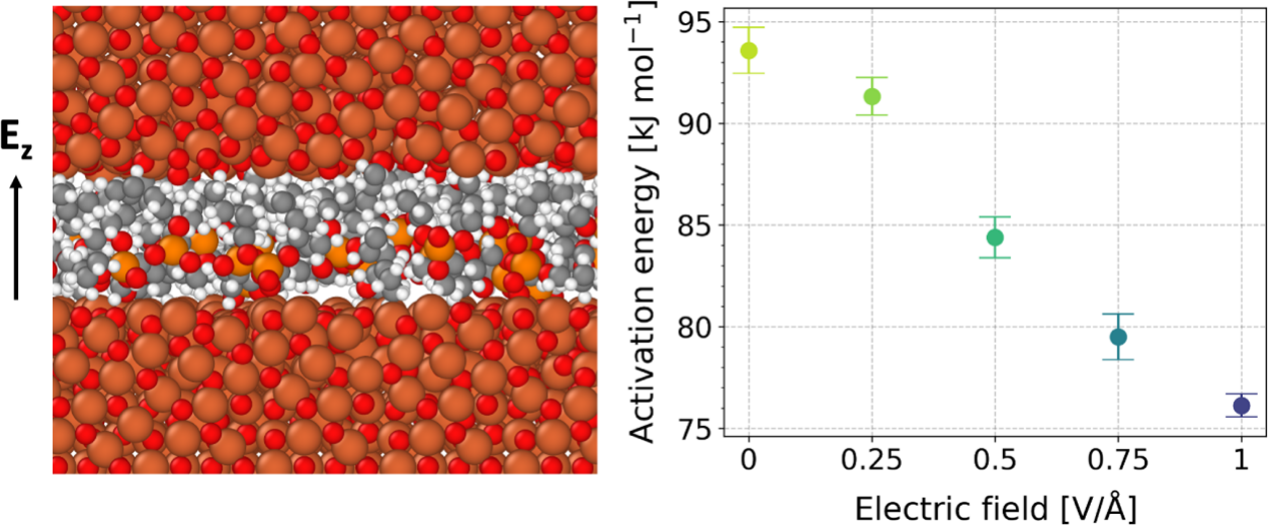

Phosphate esters decompose on metal surfaces and form protective polyphosphate films. For many applications, such as in lubricants for electric vehicles and wind turbines, an understanding of the effect of electric fields on molecular decomposition is urgently required. Experimental investigations have yielded contradictory results, with some suggesting that electric fields improve tribological performance, while others have reported the opposite effect. Here, we use nonequilibrium molecular dynamics (NEMD) simulations to study the decomposition of tri-*n*-butyl phosphate (TNBP) molecules nanoconfined between ferrous surfaces (iron and iron oxide) under electrostatic fields. The reactive force field (ReaxFF) method is used to model the effects of chemical bonding and molecular dissociation. We show that the charge transfer with the polarization current equalization (QTPIE) method gives more realistic behavior compared to the standard charge equilibration (QEq) method under applied electrostatic fields. The rate of TNBP decomposition via carbon–oxygen bond dissociation is faster in the nanoconfined systems than that in the bulk due to the catalytic action of the surfaces. In all cases, the application of an electric field accelerates TNBP decomposition. When electric fields are applied to the confined systems, the phosphate anions are pulled toward the surface with high electric potential, while the alkyl cations are pulled to the surface with lower potential, leading to asymmetric film growth. Analysis of the temperature- and electric field strength-dependent dissociation rate constants using the Arrhenius equation suggests that, on reactive iron surfaces, the increased reactivity under an applied electric field is driven mostly by an increase in the pre-exponential factor, which is linked to the number of molecule–surface collisions. Conversely, the accelerated decomposition of TNBP on iron oxide surfaces can be attributed to a reduction in the activation energy with increasing electric field strength. Single-molecule nudged-elastic band (NEB) calculations also show a linear reduction in the energy barrier for carbon–oxygen bond breaking with electric field strength, due to stabilization of the charged transition state. The simulation results are consistent with experimental observations of enhanced and asymmetric tribofilm growth under electrostatic fields.

## Introduction

Phosphate esters have many industrial applications, such as nuclear fuel processing,^1^ flame retardants,^2^ corrosion-resistant metal coatings,^3^ and lubricant additives.^4^ The primary role of phosphate esters in lubricants is to reduce the wear of rubbing metal surfaces through adsorption, molecular decomposition, and the growth of protective polyphosphate tribofilms.^4^ The molecular mechanisms of phosphate esters’ antiwear action are now relatively well understood at high temperatures and stresses, as found inside engines and transmissions.^5^ Comparatively little is known regarding their molecular decomposition and tribofilm growth in the presence of electric fields. In many modern applications, electric fields may be present across tribological contacts, for example lubricants and greases for electric vehicles (EVs)^6−8^ and wind turbines.^9,10^ Investigation of this effect is important because it could be a driver for premature failure of bearings in EVs^11^ and wind turbines.^12^ On the other hand, ready availability of electrical energy in these applications provides an opportunity for its use to promote desirable processes, such as tribofilm growth, or to suppress undesirable ones, such as corrosive wear.^13^

Several reviews have been compiled that discuss the effects of electric fields on lubrication.^13−16^ For certain types of potential future lubricants, such as ionic liquids, friction can be carefully controlled by the application of electric fields.^17^ However, experimental results for production lubricants, such as phosphate esters and zinc dialkyldithiophosphate (ZDDP) have yielded contradictory results.^13^ It was shown that ZDDP films could be grown on metal surfaces from a base oil without rubbing and at relatively low temperatures by the application of large voltages across the contact.^18^ It was also shown that ZDDP tribofilms formed under rubbing with an applied voltage were effective in reducing both friction and wear compared to when no voltage was applied.^19^ It was found that ZDDP was most effective at highly oxidizing electrode potentials, at which voltammetry showed that it formed disulfide.^20^ A more recent study of ZDDP solutions in a blend of polycarbonate and diethyl succinate also found friction and wear to be decreased at oxidizing potentials.^21^ Applying a voltage across contacts lubricated by a base oil containing dilauryl hydrogen phosphate (DHP) could significantly reduce friction.^22^ It has also been reported that the application of a voltage across a tricresyl phosphate (TCP) lubricated contact can promote film formation.^23^ However, excessive promotion of surface film formation on the cathode surface by the applied voltage decreased scuffing resistance by preventing film formation on the anode surface.^23^ Similar results have also been observed for ZDDP, where the application of an electric field across the contact reduced the wear of the cathode surface only and had a negligible effect on friction.^24^ Moreover, the application of a direct current across contacts lubricated by fully formulated transmission fluids and gear oils generally lead to increased wear and have only a minor effect on friction.^25,26^

One tool that could help to clarify the different behaviors seen experimentally under applied electric fields is molecular simulation. In particular, nonequilibrium molecular dynamics (NEMD) simulations have been used to study the influence of electric fields on a wide range of systems and processes.^27^ Dissociation processes under applied electric fields have been studied with classical force fields,^28−30^ reactive force fields (ReaxFF),^31−37^ and ab initio^38−42^ based NEMD simulations. Previous studies have highlighted the importance of the choice of the charge equilibration method in predicting physical and realistic simulation results from NEMD simulations with electric fields.^36,43−45^ The thermal^46−48^ and mechanochemical^49−53^ decomposition of phosphate esters has also been studied with ReaxFF and ab initio methods. However, the dissociation of phosphate esters has not yet been studied under applied electric fields in NEMD simulations.

In this study, we investigate the effect of electrostatic fields on the high-temperature decomposition of tri-*n*-butyl phosphate (TNBP) using NEMD simulations with ReaxFF. To study the effect of the charge equilibration algorithm, we compare the charge equilibration (QEq)^54^ and the charge transfer with polarization current equalization (QTPIE)^55^ methods. The QTPIE method enables more accurate modeling of intramolecular interactions and intermolecular charge distributions under an applied electric field.^44^ We simulate a bulk TNBP system as well as molecules nanoconfined between iron and iron oxide surfaces to investigate the influence of catalytic surfaces. Single-molecule nudged-elastic band (NEB) calculations^56,57^ are used to determine the minimum energy path for the main dissociation mechanisms on iron and iron oxide with and without an external electrostatic field. The overall aim is to provide several important molecular-scale insights into the influence of electrostatic fields on the decomposition of lubricant additives.

## Methods

### Molecular Simulation Details

The primary alkyl phosphate ester TNBP was selected due to its intermediate thermal stability between secondary/tertiary alkyl and aryl phosphate esters.^47,58^ TNBP has been used as a vapor-phase lubricant in its pure form^59^ and a lubricant additive dissolved in a base oil,^60^ for example in aviation hydraulic fluid.^61^

All simulations were performed in the large atomic/molecular massively parallel simulator (LAMMPS) software.^62^ Velocity Verlet^63^ integration was employed with a time-step of 0.25 fs.^46^ We used the version of ReaxFF^64^ implemented in LAMMPS.^65,66^ ReaxFF was originally developed to study hydrocarbon reactions,^64^ but it has now been extended to a very wide range of systems and processes.^67^ We used the ReaxFF parameters developed by Khajeh et al. for C/H/O/Fe/P systems.^46^ This ReaxFF parameter set was successfully applied to study the thermal^47,48^ and mechanochemical^50,52,53^ decomposition of several phosphate esters on iron and iron oxide surfaces. The adsorption energy of TNBP on ferrous surfaces, as well as the reaction energy and energy barrier of the main decomposition pathways (C–O and P–O dissociation), was validated against density functional theory (DFT) for these ReaxFF parameters using NEB calculations.^47^ We compared bulk and confined systems, which were constructed using the Materials and Process Simulations (MAPS) platform developed by Scienomics SARL.

### Systems Studied

#### Isolated TNBP Molecule

We first investigated the effect of electric fields on an isolated TNBP molecule. Atomic positions and charges were taken in the first time step following a conjugate gradient (CG) energy minimization. The electric force was included in the system potential energy during minimization. The dipole moment was calculated from the atomic positions and charges:
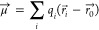
1where, *q*_*i*_ is the atomic charge of *i*^*th*^ atom, and  refers to the relative position vector from the molecular center to a reference point. The electrostatic potential was derived from the atomic charges using the Multiwfn software.^68^

A set of short MD simulations (100 ps) was run to study an isolated TNBP molecule under an applied electric field. The dipole of the molecule was initially orientated at a 20° angle to the external electric field. The atomic charges and positions were recorded at a frequency of 1 ps. The center-of-mass molecular velocity, derived from the projection of atomic velocities, was used to calculate the translational kinetic energy. The rotational energy was calculated from the molecular angular velocities. The vibrational energy was obtained from the residual atomic motions.

The change in bond lengths was computed from the atomic positions and presented with respect to the reference case without the electric field:
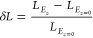
2where  and  are the bond lengths with and without an electric field, respectively.

#### Bulk System

Bulk TNBP systems were constructed as a baseline against which to compare the confined systems. The bulk systems are shown in Figure S1. For the bulk system, 48 TNBP molecules were randomly inserted into a simulation box at an initial density of 0.3 g of cm^–3^. In the current study, we do not consider the effects of base oil molecules on adsorption and decomposition.^51,52^ Periodic boundary conditions were used in all three Cartesian directions. After an energy minimization, the system was equilibrated using the isothermal–isobaric (NPT) ensemble at 300 K and 0.1 MPa, which resulted in a liquid density of TNBP in agreement with the experiment (1.0 g cm^–3^). A global Nosé–Hoover^69,70^ thermostat and barostat were used for the bulk simulations, with a damping coefficient of 25 ps for the temperature and 250 ps for the pressure. Another equilibration was then run for 0.1 ns in the canonical (NVT) ensemble. The temperature was then increased to the target value (1000–1350 K) instantaneously, the electric field was applied simultaneously to the system, and the production NVT simulation was run for 1 ns. Previous experiments suggested that TNBP begins to thermally decompose at around 530 K.^71,72^ The higher simulation temperatures were chosen to facilitate sufficient decomposition events in the available simulation time. The simulated temperatures were representative of those that may be encountered by TNBP during its application as a vapor-phase lubricant (>700 K)^59^ or an extreme pressure additive (>1000 K).^73^ The boiling point of TNBP was 566 K, and since the bulk systems were not allowed to expand during the production runs, the baseline case included the effects of spatial confinement but without the catalytic surfaces. Additional tests with the TNBP molecules in the gas phase with an NPT run at 600 K, followed by NVT simulations at the target temperature, gave much lower dissociation rates.

#### Confined Systems

For the confined systems, the TNBP molecules were placed between two ferrous surfaces. The outer layers of the steel surfaces used in tribological contacts are chemically heterogeneous and are covered by multiple different iron oxides, including FeO, Fe_2_O_3_, and Fe_3_O_4_.^74,75^ In most cases, the dominant oxide found inside the wear track is Fe_3_O_4_.^76,77^ When rubbing under high contact pressures under ultrahigh-vacuum (UHV) conditions, oxide layers of the ferrous surface can be removed, exposing nascent Fe surfaces.^78^ Due to the limited length-scales that can be simulated, most NEMD simulations adopt a single surface type for each simulation.^52,53^ Here, we compare two surfaces, α-Fe(110)^79^ and Fe_3_O_4_(001).^80^ Previous simulations have shown that TNBP is more reactive toward iron surfaces than iron oxides.^47,52^ At low temperatures (*T* < 1185 K), the nascent Fe surface of the body-centered cubic crystal structure (α-Fe) is the most stable, while at high temperatures (1185 K < *T* < 1667 K), the face-centered cubic lattice (γ-Fe) is favored. Therefore, γ-Fe(111) is also considered for the high-temperature confined NEMD simulations.^79^

The ferrous surfaces had approximate dimensions of 5.0 nm in the *x*- and *y*-directions, with a thickness of 1.1 nm for the Fe_3_O_4_ surface and 0.8 nm for the Fe surfaces in the *z*-direction. Initially, the two surfaces were separated by 3.0 nm of vacuum in the *z*-direction. 48 TNBP molecules were inserted between two ferrous surfaces, giving a surface coverage of approximately 1 molecule nm^–2^. Half of the TNBP molecules were orientated with P=O bonds pointing along the positive *z*-direction, while the other half were pointed in the opposite direction.^47^ This surface coverage and initial orientation were chosen to mimic the monolayer adsorption of TNBP, as observed experimentally for phosphate esters from the vapor phase^81^ and from base oil solution.^82^

**Figure 1:**
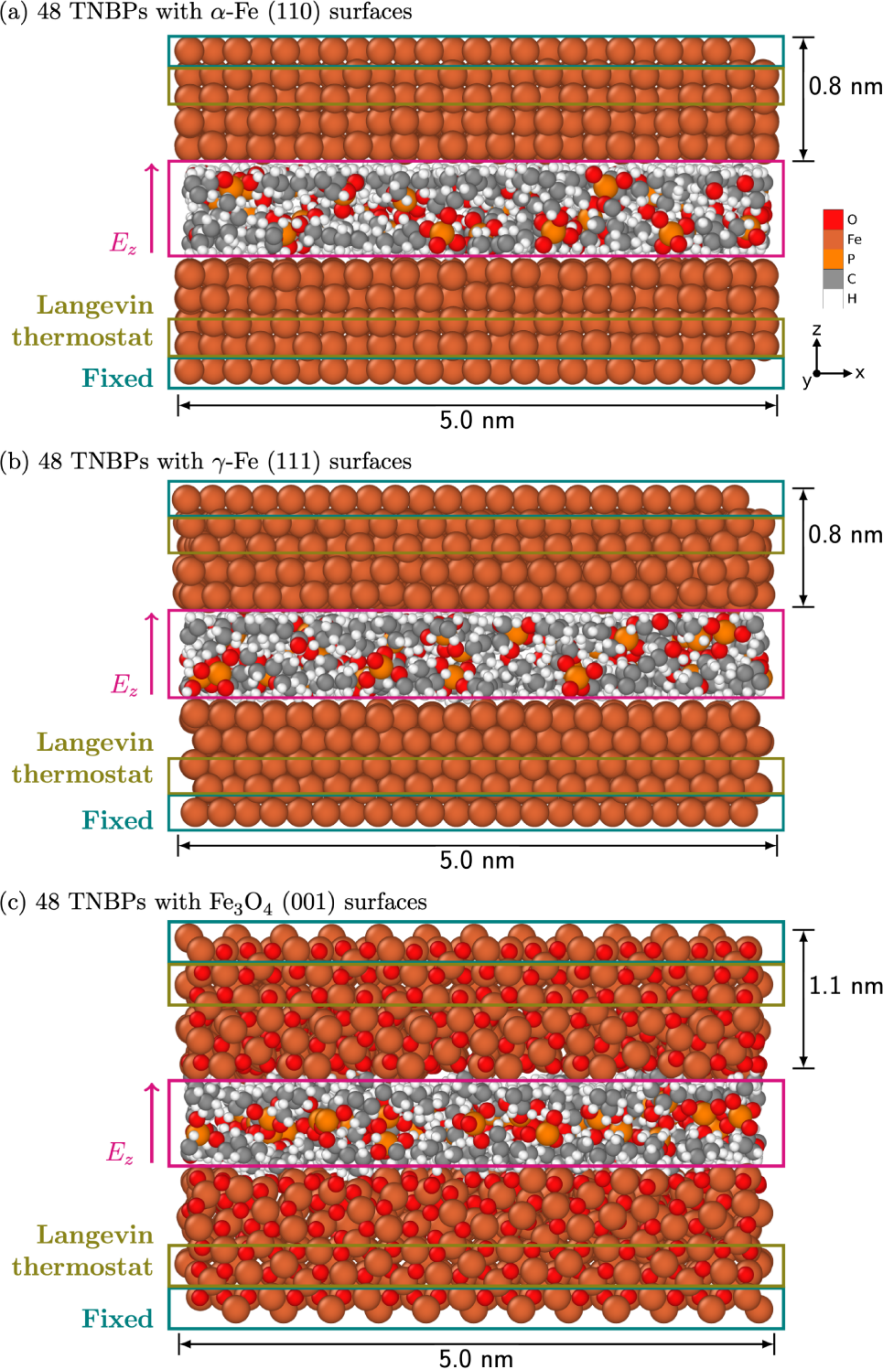
Snapshots from simulations containing 48 TNBP molecules confined between (a) nascent α-Fe(110) surfaces under 1200 K, (b) nascent γ-Fe(111) surfaces above 1200 K, and (c) Fe_3_O_4_(001) surfaces for all temperatures, after energy minimization and equilibrium.

For the confined systems, a fixed boundary was used in the *z*-direction and periodic boundary conditions were applied in the *x*- and *y*-directions. The system was first energy minimized, and then, the top surface was moved downward at a velocity of 10 ms^–1^ until the density of the TNBP region reached 1.0 g cm^–3^. The lowermost layer of atoms in the bottom surface was fixed in the *z*-direction. The system was then equilibrated at 300 K and 0.1 MPa for 0.2 ns. Atmospheric pressure was maintained by applying a constant normal force to the topmost layer of atoms of the top surface. Snapshots of the systems after energy minimization and equilibration are presented in Figure 1. After equilibration, the positions of the atoms of the outer layer on the top surface were fixed in the *z*-direction. The temperature was then instantaneously raised to its target value, and an electrostatic field was simultaneously applied along the positive *z*-direction. A similar approach was successfully applied to capture the combined effect of temperature and shear stress in previous NEMD simulations.^50,52^ These production simulations under an electric field were performed for 1.0 ns. The temperature in the NEMD simulations was controlled with a Langevin thermostat.^83^ The thermostat was applied only to a middle layer of atoms in the surfaces, as shown in Figure 1. It takes some time for the heat to transfer from the surfaces to the molecules and the temperature in the center of the system to reach the target value. This process takes about 40 ps for a typical simulation, so the heating rate is around 17.5–25 K ps^–1^ (depending on the target temperature), which is similar to those used in previous ReaxFF simulations.^84^

### NEB Calculations

The NEB calculations were performed in LAMMPS^62^ with the same ReaxFF parameters as those used in the NEMD simulations.^46^ The systems for the NEB calculations consisted of a single TNBP molecule adsorbed on the same ferrous surfaces used in the NEMD simulations. Minimum-energy reaction paths for both the C–O and P–O bond cleavage were calculated with and without electric fields using the climbing image scheme.^56,57^ A total of 20 replicas were used, including the initial and final replicas. The intermediate replicas were created via linear interpolation. A spring constant of 400 kcal mol^–1^ Å^–2^ was applied to connect the adjacent replicas along the transition path. The electric potential energy was included in the total system potential energy during the NEB calculations.

### Charge Equilibration Methods

Energies calculated in ReaxFF molecular dynamics simulations are described by eq 3:^65^
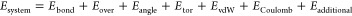
3where, *E*_bond_ is the bond energies obtained from bond order BO_*ij*_. BO_*ij*_ is calculated from the interatomic distance and is contributed by σ bonds, π bonds, and π–π bonds. BO_*ij*_ corrections are often necessary in covalent systems to eliminate weak binding interactions with nonbonded neighboring atoms. *E*_over_ adds an energy penalty for overcoordinating atoms. *E*_angle_ and *E*_tor_ are both BO_*ij*_-dependent terms, referring to the three-body angle and four-body torsional angle strain, respectively. *E*_vdW_ and *E*_Coulomb_ represent the electrostatic and dispersive interactions between all atoms in the system, regardless of their connectivity and BO_*ij*_. *E*_additional_ considers system-specific terms such as lone pair energy and torsion conjugation.

*E*_Coulomb_ is calculated from the interatomic Coulomb force, which is directly related to the atomic charges for each atom pair. The electric field present in the system distorts the electron cloud of a molecule, and atomic charges are perturbed due to molecular polarization. Under the applied electric field, atoms in ReaxFF simulations experience a force from the electric field:
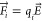
4which is proportional to the atomic charge *q*_*i*_ under an electric field . Note that *E* with an arrow above or the subscripts *x*, *y*, or *z* denote an electric field, rather than energy. In ReaxFF-based molecular dynamics simulations, the atomic charges continuously vary during the simulation in response to the local chemical environment. Most previous simulations with ReaxFF calculate the atomic charges using variants of the electronegativity equalization method (EEM)^85^ or the QEq method.^54^ In the QEq method, along with the electroneutrality constraint (), the atomic charges are updated at each time step by minimizing the electrostatic energy (eq 5):^54^
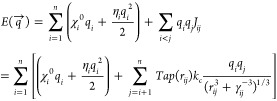
5where the coefficients  and η_*i*_ represent the Mulliken electronegativity^86^ and the Parr–Pearson hardness,^87^ respectively. *q*_*i*_ refers to the charge of the *i*^th^ atom, *J*_*ij*_ is the screened Coulomb interaction, *Tap*(*r*_*ij*_) is a distance-dependent 7^th^ order polynomial to avoid discontinued energy, *k*_c_ is the dielectric constant, and γ_*ij*_ is a shielding parameter for *i*^th^ and *j*^th^ atoms. With the application of an electric field, some studies^31,88,89^ have implemented the modified electronegativity () proposed by Chen and Martínez.^90^ However, this approach is insufficient to describe polarization for systems of many molecules or isolated large molecules, due to the long-range charge transfer used in the QEq method.^37^ This leads to unrealistic spatially dependent charge distribution and therefore unphysical translational movement under electric fields.^36,55,91^ Therefore, this implementation is not studied here.

To correctly predict the charge distribution in molecular dynamics simulations with ReaxFF, various modified charge equilibrium methods have been proposed, including the split charge equilibration (SQE) method,^92^ the QTPIE method,^55^ and atom-condensed Kohn–Sham density functional theory approximated to second order (ACKS2) method.^93^ These modified charge equilibrium methods address, up to a certain accuracy, the shortcoming of the QEq method, which performs long-range charge transfer. Therefore, the Coulomb interaction is restricted to local neighboring atoms. ACKS2 is an extension of the EEM, but the charges are derived by DFT at the atomic scale. ACKS2 restricts the long-range charge transfer and correctly describes the charge distribution at the dissociation limit.^93^ The atomic chemical potential in molecular dynamics simulations using ACKS2 considers energies calculated from the EEM approach and the Kohn–Sham formulation. The Kohn–Sham kinetic energy is interpreted as Legendre transform and extended to second order in ACKS2.^93^ However, under an external electric field, for a system larger than the Coulomb cutoff distance, the short-ranged restraint in ACKS2 could result in inaccurate field screening.^36^ The ACKS2 method also requires reparametrization of the ReaxFF potential, which limits its wide transferability. Currently, it has only been applied in ReaxFF-based molecular dynamics simulations of a few systems, such as water^36^ and lithium–oxygen systems.^94^

In the QTPIE method,^55^ the partial charge *q*_*i*_ is extracted from a polarized current *p*_*ji*_ (). The polarized current defines a migration tendency of the electronic density between two atoms, enabling a description of in-plane polarizabilities. Using the concept of charge transfer with polarization current, the electrostatic energy in eq 5 can be rewritten as eq 6:^55^


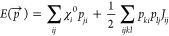
6These partial charges in the bond space are transformed into atomic charges in the atom space by replacing the electronegativity  in eq 6 with an effective electronegativity χ_eff,*i*_:^91^
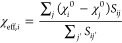
7where *S*_*ij*_ is the overlap integral of the atomic orbitals of *i*^*th*^ and *j*^*th*^ atoms. The idea of effectively shielding long-range charge transfer by using the overlapping charge distributions has also been adopted in the polarizable charge equilibration model (PQEq).^95^ The QTPIE method penalizes the long-range charge transfer of the QEq method by introducing a distance decay function:
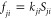
8where *k*_*ji*_ is a charge-independent scaling factor.^55^ Substituting eqs 7 and 8 in eq 6, the electrostatic energy described in the QTPIE method is
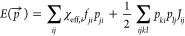
9In the presence of an electric field, the effective electronegativity χ_eff,*i*_ in eq 9 has been further amended as
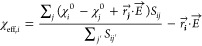
10The implementation of the effective electronegativity is origin invariant, and the molecular polarization is spatially independent under electric fields.^37,96^ The details are shown in eq S1. Therefore, the QTPIE method enables periodic systems under applied electric fields. Following a similar formulation as the QEq method, implementing the QTPIE method in MD simulations does not require reparametrization of the ReaxFF potentials. Gergs et al. and Kritikos and Giusti have successfully demonstrated realistic molecular polarization and charge distribution under electric fields in ReaxFF NEMD simulations, using the QTPIE method for charge equilibration.^43,44,97^ Therefore, in this study, the QTPIE method has been selected to compare with the standard QEq method. We use the implementation of QTPIE in LAMMPS due to Kritikos and Giusti.^44^

For all of the NEMD simulations in this study, an electrostatic field was applied uniformly along the positive *z*-direction (*E*_*z*_ = 0.25–1.00 V/Å). This approach mimics experiments in which an electric field is applied across a tribological contact.^25,26^ The strength of the electric fields used in our NEMD simulations is higher than the maximum typically applied in nanoscale tribology experiments using atomic force microscopy (∼0.1 V/Å)^98^ and macroscale tribology experiments under elastohydrodynamic lubrication conditions (∼0.02 V/Å) with a minimum film thickness between 50 and 1000 nm.^99^ This is a common limitation of NEMD simulations,^27^ and similar field strengths have also been adopted by previous ReaxFF studies.^32,34,100^ In this study, a high field strength is required to ensure that a sufficient number of the decomposition processes of the lubricant additives have occurred within a reasonable time (normally nanoseconds) in the ReaxFF NEMD simulations, which are much shorter than tribology experiments (usually minutes).^25,26^ Electric field strengths of the order of 1 V/Å are commonly required to promote bond dissociation.^101,102^ Moreover, in macroscale tribology experiments under boundary lubrication conditions,^25,26^ the exact film thickness is not known. For the multiasperity rough surfaces, there are likely to be some areas, where the asperities on opposing surfaces are close to contact, and the film thickness is very small (Å-scale). Thus, under applied voltages of around 1 V, film thicknesses comparable to those used in our NEMD simulations can be expected.

### Decomposition Reaction Analysis

Phosphate esters decompose and form iron phosphate or polyphosphate tribofilms on rubbing ferrous surfaces, which are responsible for their antiwear performance.^103^ These protective films can also be formed thermally without rubbing, although high temperatures (∼800 K) are required.^58,104^ It has been outlined that the decomposition mechanisms of phosphate esters are mainly through the C–O and P–O bond cleavage.^47,58^ For TNBP and other alkyl phosphates, C–O bond dissociation is the rate-determining step for the thermal decomposition process.^47,50^ Therefore, we focus our rate analysis on C–O bond cleavage. Atomic trajectories and bonding information with a bond order cutoff of 0.3 were recorded at a frequency of 1.0 ps.

## Results and Discussion

### Isolated TNBP Molecule

**Figure 2:**
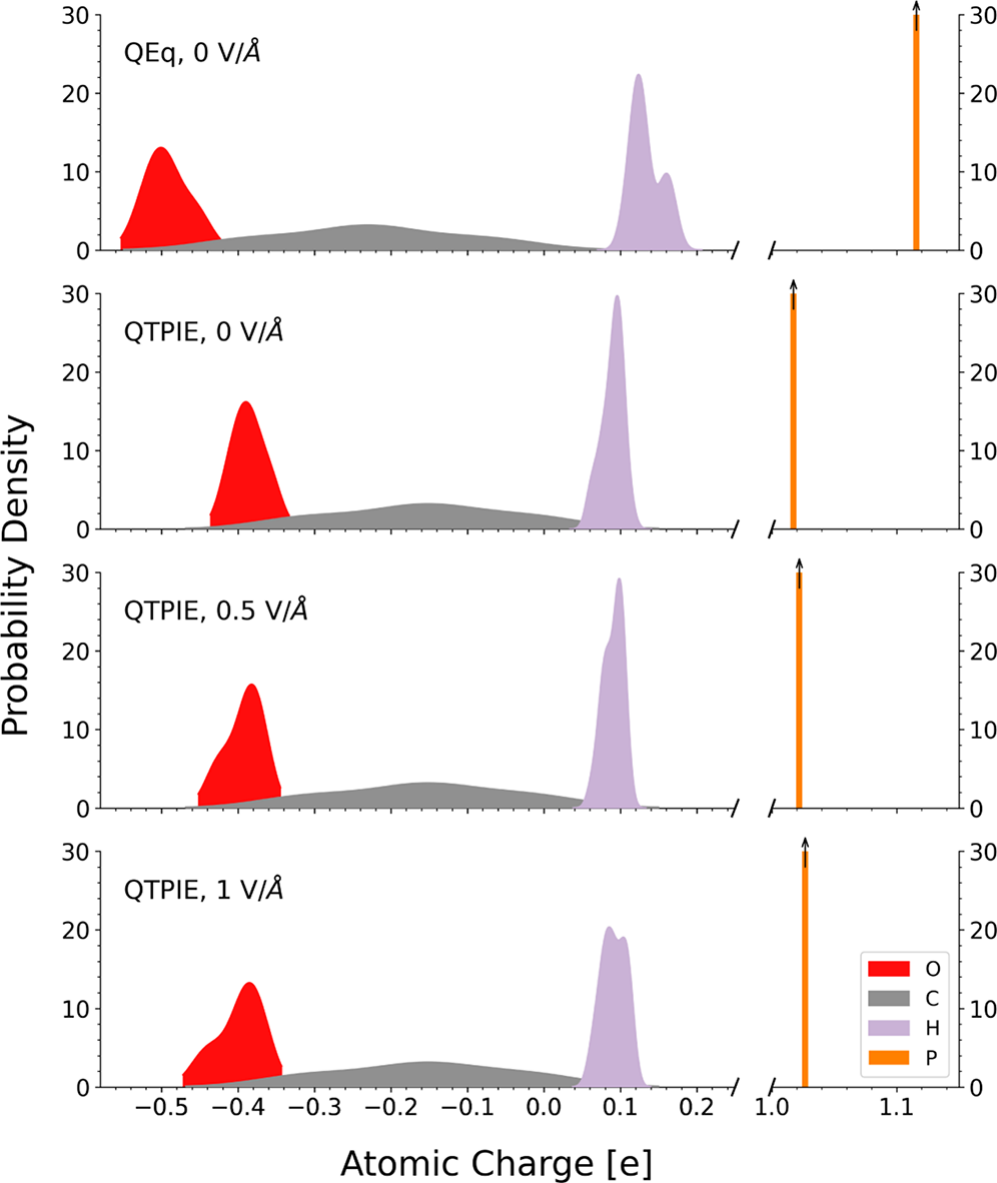
Atomic charge distribution under electric fields for a minimized isolated TNBP molecule using the QEq method and the QTPIE method.

First, we compare the two selected charge equilibration methods, QEq and QTPIE. Atomic charges of an isolated TNBP molecule under an electric field are shown in Figure 2. Before the electric field is applied, the charges predicted by the QEq method are approximately one-third higher than those predicted using the QTPIE method. Similar overestimation of the atomic charges for QEq has also been noted for other molecules.^105^ The carbon and oxygen atoms carry more negative charges with QEq than those with QTPIE, while the hydrogen and phosphorus atoms are more positive. This is due to the long-range charge transfer strategy adopted by the QEq method. The lower atomic charges predicted by the QTPIE method demonstrate the shielding effect of long-distance charge transfer.^55^ Since the applied electric field does not alter the charges in the QEq method, only the reference case (QEq, 0 V/Å) is shown.^44^ Using the QTPIE method, a wider distribution of atomic charges is observed, as the field strength is increased, which is due to electric field-induced polarization.^55^

**Figure 3:**
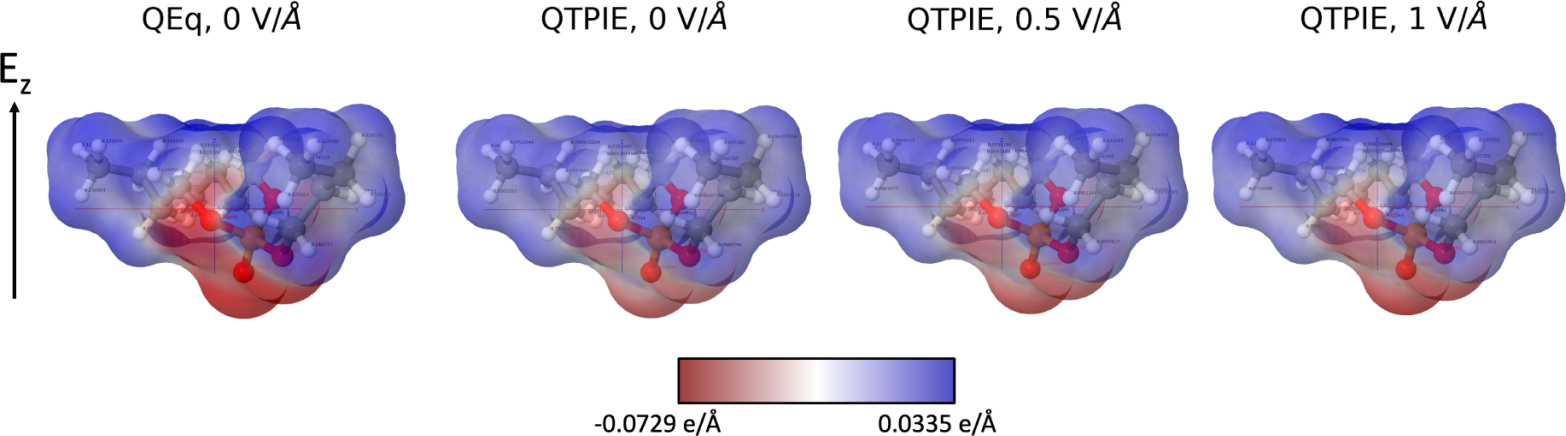
Electrostatic potential of a TNBP molecule under different electric fields in multiples of electron charge, colored on its accessible surface area.

Figure 3 presents electrostatic potentials of the TNBP molecule on its accessible surface area. Comparing the results obtained with the QEq and QTPIE methods for the case with no external field, one can note some differences in the atomic charge distribution, which arise from the shielding of intramolecular interactions in the QTPIE method. With QEq, the same charge distribution is observed for all of the cases, since the presence of the external field does not affect the charge equilibration process. The effects of the external electrostatic field on the charge distribution are evident, when the QTPIE method is used with the polarization that follows the direction of the electric field and increases with increasing field strength.^44^ Using the QTPIE method, in response to the electric field, the charge distribution within the molecule changes, and simultaneously, the molecular dipole moment aligns with the direction of the field (Figure S2). In the absence of an electric field, the dipole moment is 3.17 D when using the QTPIE method and 5.38 D when using the QEq method. For reference, the experimental value of the permanent dipole moment of pure TNBP is 3.32 D,^106^ and classical force fields, such as the general Amber force field (GAFF) and modified versions, thereof give a dipole moment of 2.90–3.58 D.^107^ The difference in the atomic charge distribution within an isolated large molecule, observed between the QEq method and the QTPIE method, is due to the shielding of the long-range intramolecular charge transfer in the QTPIE method. The accumulated errors of the higher atomic charges in the QEq method lead to the overestimated dipole moment, particularly for highly polar molecules.^55^ Under an applied electric field, the dipole moment remains constant for QEq but increases with increasing field strength up to ∼5 D at 1.0 V/Å, as shown in Figure S2.

**Figure 4:**
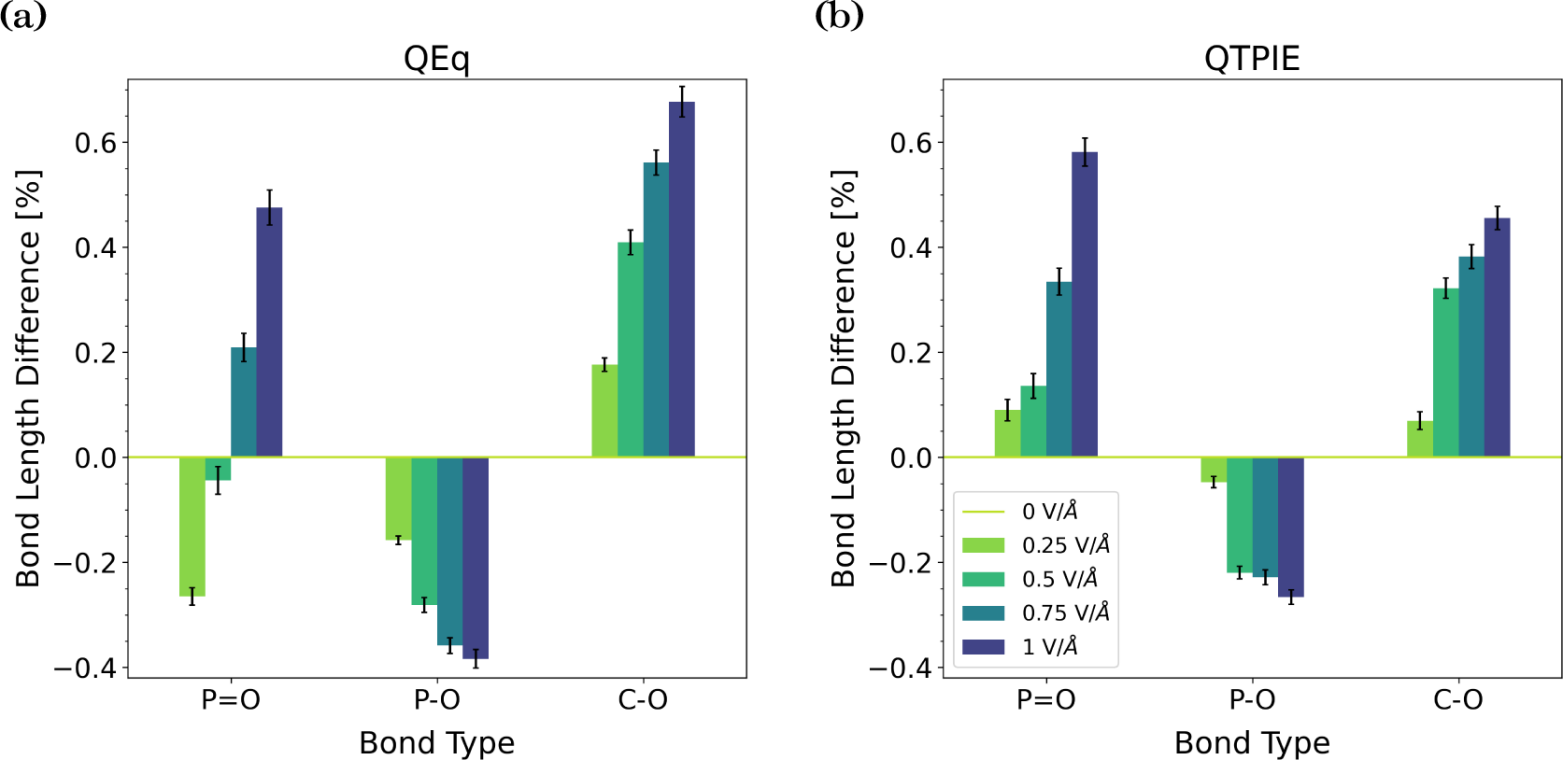
Change in the bond lengths of the isolated TNBP molecule in the NEMD simulations under applied electric fields using (a) the QEq method and (b) the QTPIE method.

Using the QTPIE method, electric field-enhanced polarization aligns the molecular dipole with the field direction. This alignment increases the spatial separation of the electronegative oxygen atoms from the other atoms. As a result, the P=O bond and the C–O bond were stretched, while the P–O bonds were slightly compressed. Elongation of C–O bonds under applied electrostatic fields has also been observed for other polar molecules using DFT.^108^ As shown in Figure 4, with QTPIE, the percentage changes in the bond lengths increase with an increasing field strength. The QEq method exhibits stronger bond stretching of the C–O bonds and P–O bond compression under applied electric fields, which is due to the higher atomic charges. At low field strengths (≤0.5 V/Å), the P=O bonds are also compressed with QEq, whereas at higher field strengths, they are stretched as with QTPIE.

### Bulk Systems

**Figure 5:**
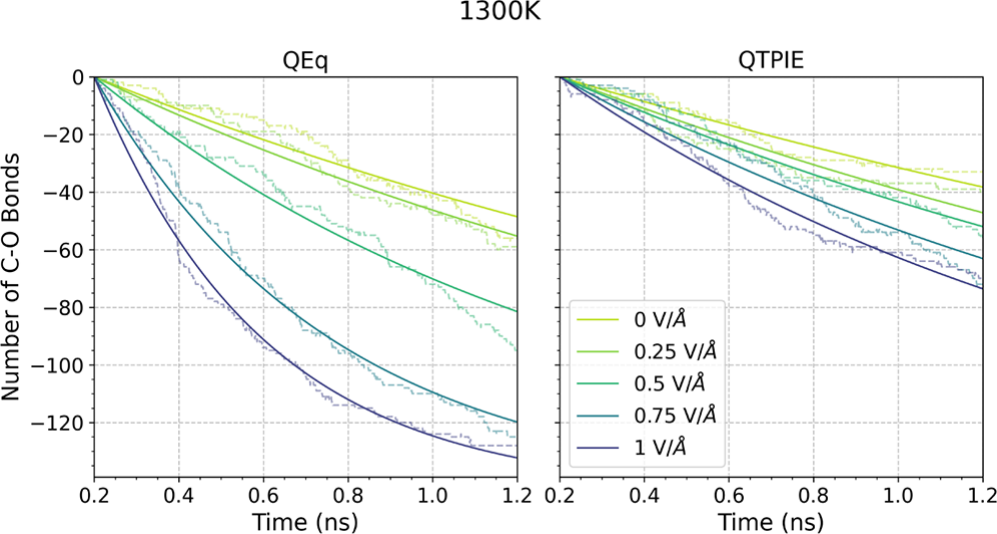
Change in the number of C–O bonds for the bulk TNBP system at 1300 K under different external electric field strengths by using the QEq method and the QTPIE method.

The effect of charge equilibration on TNBP decomposition under an applied electrostatic field at 1300 K is shown in Figure 5. In the absence of the electric field, the change in the number of C–O bonds in the TNBP molecules with time was similar between the QEq and the QTPIE methods. This implies that QEq is suitable to study TNBP dissociation when no external electric field is applied.^50^ Under the applied electric fields, a significant enhancement of the bond dissociation was observed using the QEq method. While the same accelerated dissociation is also observed with QTPIE, the effect of the electric field on the reactivity is not as pronounced as when using the QEq method. This is due to the higher atomic charges predicted by the QEq method compared to those predicted by the QTPIE method. Under an equivalent electric field, referring to the eq 4, the force is proportional to the atomic charge. Therefore, the electrostatic force acting on the atoms is higher using the QEq method than that using the QTPIE method. This leads to increased stretching of the C–O bonds for QEq compared to QTPIE (Figure 4), which makes them more acceptable to dissociation. Overestimated reactivity was also observed using the QEq method in the confined systems under external electric fields (Figure S3).

**Figure 6:**
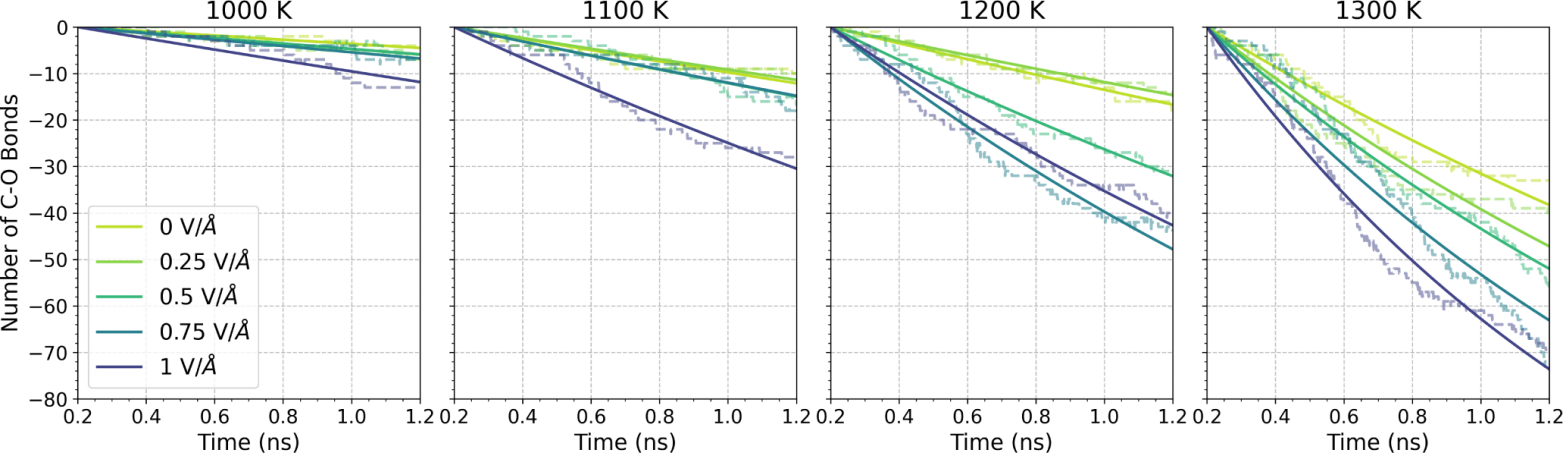
Change in the number of C–O bonds for the bulk systems containing 48 TNBP molecules at different temperatures and under electric field strengths.

Since we have shown that QEq is unsuitable to study our system under applied electric fields, we use the QTPIE method for the remainder of this study. The change in the number of C–O bonds with time at different temperatures and under electric fields for the bulk system using QTPIE is shown in Figure 6. Without an applied electric field, only 5 C–O bonds were broken during the simulation period at 1000 K. As the temperature increased, C–O cleavage was enhanced, and at 1300 K, approximately 40 out of the total 144 C–O bonds were broken (∼30%). Under the applied electric fields, an increased C–O cleavage was observed at all temperatures. In all cases, there is an exponential decay in the number of C–O bonds in the TNBP molecules, which is indicative of first-order kinetics.^50^ The solid lines shown in Figure 6 are used to determine the first-order rate constants for C–O dissociation (Figure S4).

### Confined Systems

**Figure 7:**
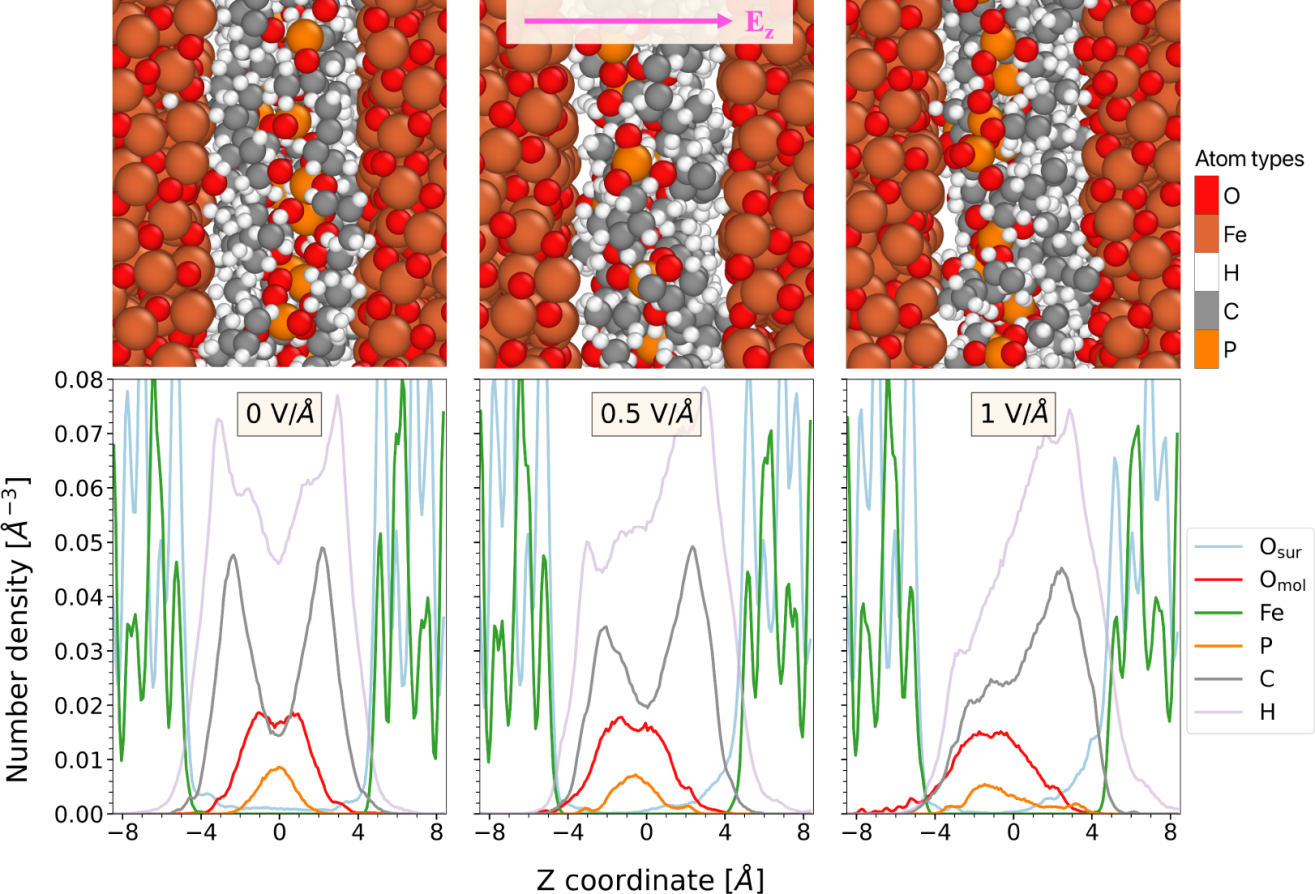
Simulation snapshots of TNBP molecules confined between two Fe_3_O_4_ surfaces at 1100 K under different electric field strengths (top) and corresponding through-thickness number density profiles for different atom types (bottom). For both figures, the electric field was applied along the positive *z* direction, as shown by the pink arrow.

Snapshots of the TNBP systems confined between Fe_3_O_4_ surfaces are presented in Figure 7. The corresponding number density profiles are also shown for the different atom types in the system. For the reference case (*E*_*z*_ = 0 V/Å), the P and O atoms in the TNBP molecules were located in the center of the nanoconfined gap, indicating limited access to the surface. The C atoms were evenly distributed in two peaks located close to the two Fe_3_O_4_ surfaces. Significant overlap between the C and Fe atoms is observed in Figure 7. In response to the electrostatic field, the rearrangement of the TNBP molecules was observed, as the molecular dipoles were aligned along the field direction. Heterolytic cleavage of the C–O bonds in TNBP leads to the formation of phosphate anions and alkyl cations. Under the electrostatic fields, the phosphate anions were mainly located near the bottom surface with higher electric potential, while the alkyl cations were pushed closer to the top surface with lower potential. The spatial rearrangement became more pronounced as the electric field strength was increased. Electric field-induced rearrangement of dissociated water molecules between iron oxide surfaces was also noted in previous NEMD simulations.^41^ The corresponding atomic charge distributions for the Fe_3_O_4_ confined systems are shown in Figure S5.

**Figure 8:**
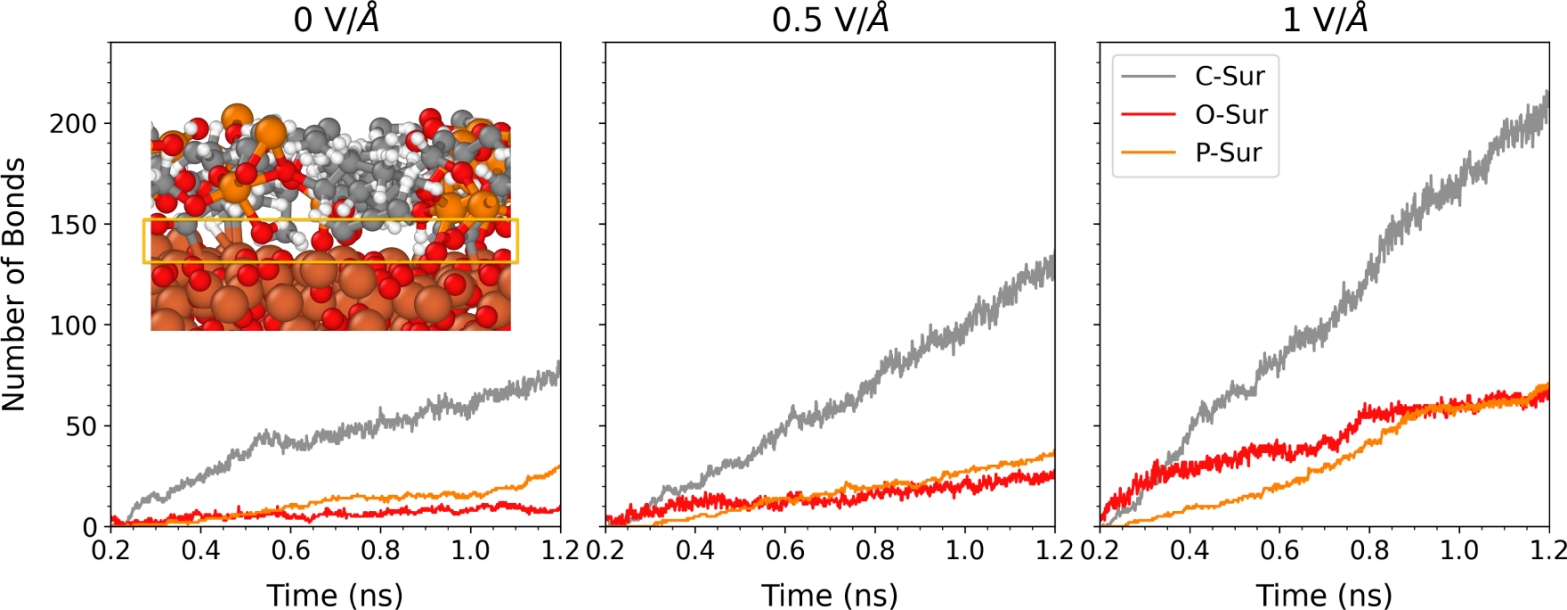
Number of covalent bonds formed between the Fe_3_O_4_ surfaces and TNBP molecules under different electric field strengths at 1100 K.

Even with no external electric field applied, the C, O, and P atoms in TNBP form covalent bonds with the surface atoms, as shown in Figure 8. As the electric field strength is increased, there are increasing numbers of C-surf, O-surf, and P-surf bonds formed between the molecules and the surface. In our simulations, the bonding becomes asymmetric, with the TNBP C atoms bonding primarily to the top surface with a lower electric potential and the O and P atoms bonding only to the bottom surface with a higher potential, as shown in Figure 7. This observation has consequences in terms of protective tribofilm formation, since for tribological systems with an electric field applied across the contact,^13^ polyphosphate formation would occur only on the surface with high electric potential, while the low potential surface would only contain a carbon-based film.^109^ Experiments have indeed shown that the chemisorption and polyphosphate tribofilm growth of ZDDP is enhanced on the positively charged surface when electric fields are applied.^21^

**Figure 9:**
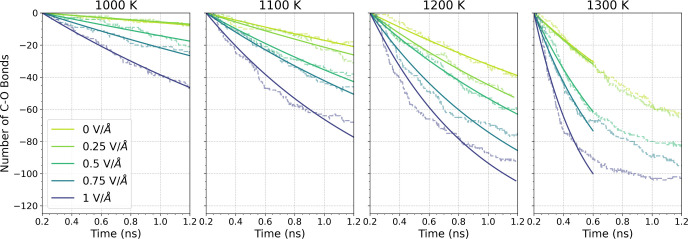
Change in the number of intact C–O bonds within TNBP molecules on Fe_3_O_4_ surfaces under different temperatures and applied electric fields. The dashed line represents the decay obtained from the MD simulations, while the solid line depicts the fitted curve of the decay using an exponential function. For simulations at 1300 K, the curve was exponentially fitted with a shorter period of time (0.4 ns).

The dissociation of the nanoconfined TNBP molecules was investigated over a range of field strengths and temperatures. Figure 9 shows the change in the number of C–O bonds with time for the Fe_3_O_4_ system. The C–O bond decay with time is fitted with an exponential function, indicating a first-order reaction.^50^ For most conditions, the first-order rate constant is calculated over the entire 1.0 ns of the simulations. For the highest temperatures (≥1300 K), the rates are calculated only for the initial 0.4 ns, because the rates saturate at longer times due to the finite number (144) of C–O bonds. As shown in Figure 9, a rate constant for each condition is extracted from the exponential fit. As expected, the reaction rate constant increased as the temperature increased. At a given temperature, the rate constant increased with an increasing electric field strength.

#### Reaction Kinetics

**Figure 10:**
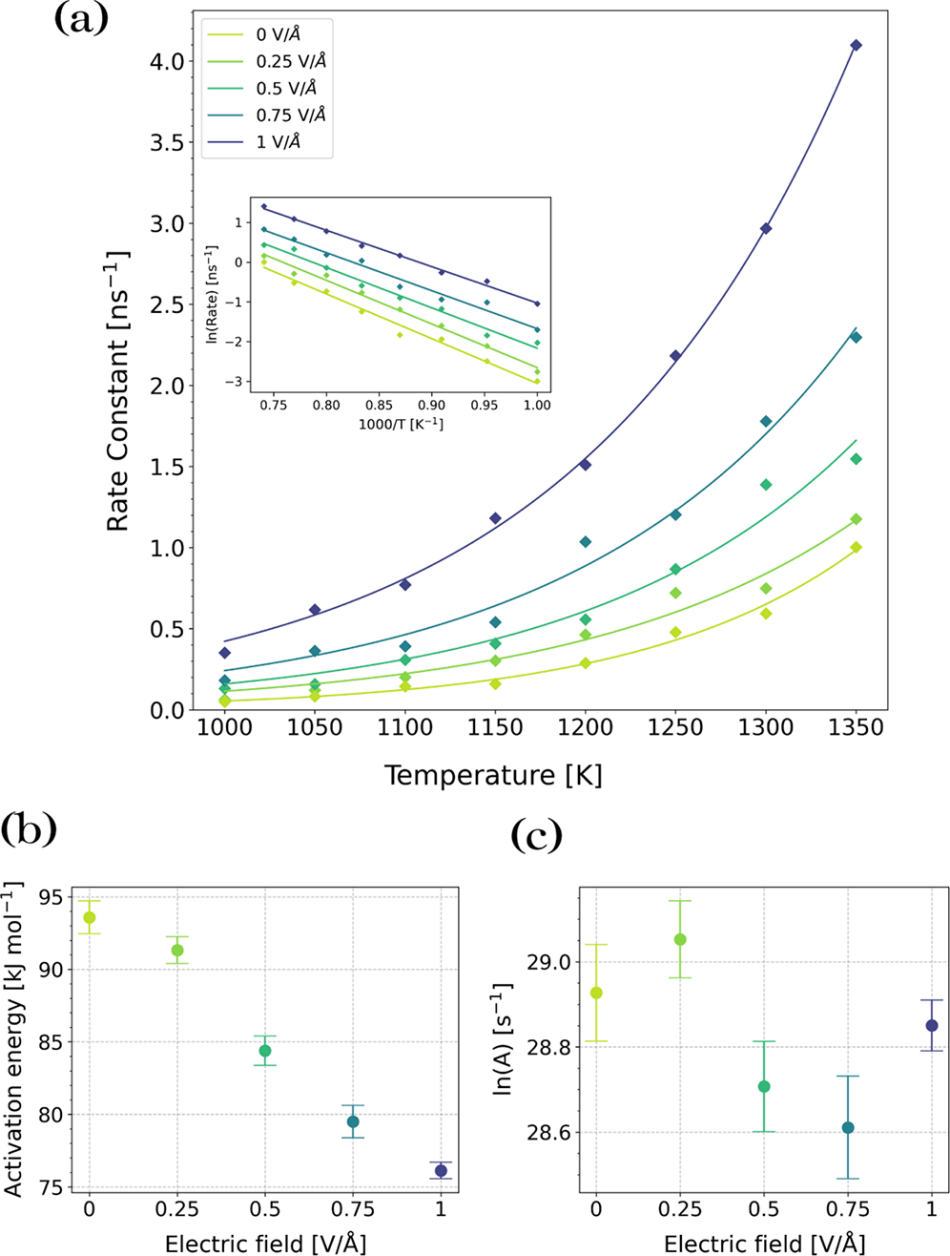
Effect of the electric fields on reaction kinetics for TNBP molecules confined between Fe_3_O_4_ surfaces, (a) combined dependence from the electric field and temperature of C–O bonds scission rate; (b) change in activation energy, *E*_a_ and (c) pre-exponential factor with external electric field strength.

The temperature-dependence of the C–O bond dissociation rate is described using the Arrhenius equation:
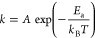
11where *k* is the rate constant for the reaction, *A* is the pre-exponential factor (or frequency factor), *E*_a_ is the activation energy, *k*_*B*_ is the Boltzmann constant, and *T* is the decomposition temperature. Rate constants under each condition were fitted to eq 11 to calculate *E*_a_ and ln(*A*), as summarized in Table S1. Figure 10a shows the change in the rate constant for C–O dissociation with temperature for the Fe_3_O_4_ system, while the inset shows the same data as an Arrhenius plot (ln(*k*) vs 1000/*T*). The slopes of the linear fits in the inset are used to calculate *E*_a_, while *A* is determined from the intercept.

From Figure 10b, the activation energy for TNBP dissociation without an external electric field was 94 kJ mol^–1^, which is in good agreement with previous glassware experiments of TNBP containing steel coupon catalysts (80 kJ mol^–1^).^72^ As the strength of the electric field was increased, the activation energy decreased to 76 kJ mol^–1^ at 1 V/Å. This observation is consistent with previous NEMD simulations of dissociation reactions under external electric fields.^28,37^ A previous experimental study also suggested that tribofilm formation was promoted by the electric field due to a reduction in activation energy for the ZDDP decomposition reaction,^21^ although this was not directly quantified. For the Fe_3_O_4_ system, there is no clear trend for the change in the pre-exponential factor with electric field strength (Figure 10c). When an electric field is applied, the dipole alignment of the polar molecules reorientates their conformation. Electric field-altered polarization (Figure 3) leads to increased separation of the electronegative O atoms and the electropositive C atoms, leading to the elongation of the C–O bonds (Figure 4). This will make the C–O bonds more labile compared with the no-field case, which is also the case for the bulk system. An additional effect for the confined system is the fact that the phosphate anions are pushed toward the high potential surface, while the alkyl cations are moved toward the low potential surface (Figure 7). Compared to the no-field case, the C–O groups are pushed closer to the catalytic Fe_3_O_4_ surface, which may further increase the reactivity. In experiments, the formation of a thicker tribofilm when electric fields were applied has been observed, which has been attributed to the reorientation of additive molecules.^110^ In summary, spatial rearrangement, molecular polarization, and increased chemisorption due to the electric field enhanced the thermal decomposition of TNBP on Fe_3_O_4_ surfaces.

Similar enhancements in the rate constant for C–O dissociation under applied electric fields were also observed for the Fe surface (Figure S6), as shown in Figure S7. As for the Fe system, *E*_a_ and ln(*A*) were calculated by fitting the temperature-dependence of the rate constant using eq 11 and are shown in Figure S8 and Table S1c. Unlike for the Fe_3_O_4_ system, *E*_a_ is not affected by the electric field strength between 0.0 and 0.5 V/Å, and it increases slightly at higher field strengths. On the other hand, ln(*A*) increases linearly with increasing electric field strength. Previous experimental studies of water dissociation on catalytic surfaces have also suggested that electric field-enhanced reactivity was also due to increases in the pre-exponential factor, rather than reductions in the activation energy.^111^ More generally, the pre-exponential factor is often found to be an important factor in electrocatalytic processes.^112^

#### NEB Calculations

**Figure 11:**
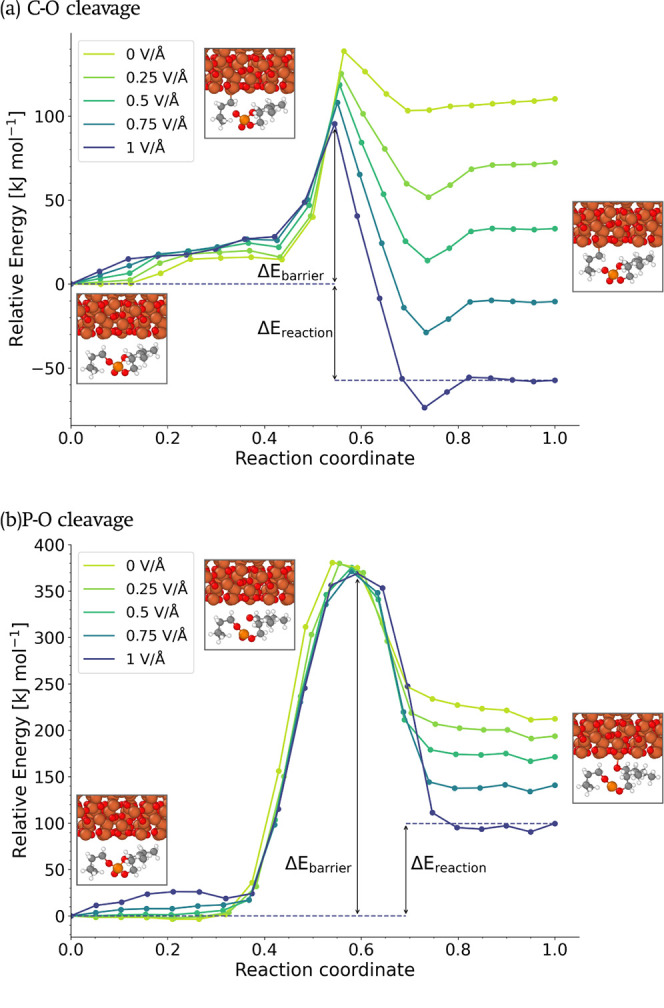
Relative energies and structures from NEB calculations for (a) C–O cleavage and (b) P–O cleavage in a single TNBP molecule on a Fe_3_O_4_ surface. The energy barrier (Δ*E*_barrier_) and reaction energy (Δ*E*_reaction_) are illustrated for the *E*_*z*_ = 1 V/Å case. The inset figures, from left to right, are the structures at the initial state, the transition state, and the final state.

We also performed NEB calculations to investigate the effect of electric fields of the first C–O and P–O dissociation reactions for single TNBP molecules adsorbed on an Fe_3_O_4_ surface. Note that the energy change for the second and third dissociation reactions may be different than that for the first, as shown in DFT calculations of the P–O dissociation reactions for trimethyl phosphite on Fe surfaces.^113^Figure 11 presents the structures and relative energies obtained from the NEB calculations, for C–O cleavage (Figure 11a) and P–O cleavage (Figure 11b) in a TNBP molecule on a Fe_3_O_4_ surface. The energies are presented with respect to the potential energy for the initial structure under each condition (Table S2). In the reference case (*E*_*z*_ = 0 V/Å), both the C–O and P–O dissociation reactions are endothermic for TNBP on Fe_3_O_4_, with reaction energies of 110 and 212 kJ mol^–1^, respectively. The energy barrier on Fe_3_O_4_ is around 140 kJ mol^–1^ for C–O dissociation and almost 400 kJ mol^–1^ for P–O cleavage. For the Fe surface (Figure S9), C–O dissociation is slightly exothermic, whereas P–O dissociation is highly endothermic, which is consistent with our previous NEB calculations.^47^ The much lower energy penalty for C–O dissociation in comparison with the P–O cleavage confirms that the former is the dominant decomposition process for TNBP on both surfaces. This observation is in agreement with previous experiments.^58^ As the applied electric field strength was increased, the energy barrier for C–O dissociation on Fe_3_O_4_ decreased, down to a minimum of 100 kJ mol^–1^ at 1.0 V/Å. The energy barrier is also decreased by increasing the electric field strength on the Fe surface (Table S3). For the unimolecular reactions described in the NEB calculations, the reduced energy barrier is dominated by the polarization of the molecules, which leads to elongation of the C–O bond under applied electric fields. The reaction energy for C–O dissociation also decreased with increasing field strength, with the reaction on Fe_3_O_4_ becoming exothermic at high field strengths (≥0.75 V/Å). These behaviors are due to electrostatic stabilization of the charged transition state and products of the dissociation reaction by the electric field relative to the reactants.^114^ Both the thermal MD simulations (Figure 10b) and the NEB calculations (Figure 11a) show that the increase in the reactivity of TNBP on Fe_3_O_4_ under external electrostatic fields is primarily due to a reduction in the energy barrier. Both with and without external electric fields, the activation energy from the multimolecular MD simulations is somewhat smaller than the energy barrier from the single-molecule NEB calculations. This is likely due to the fact that the MD simulations includes dissociation of the second and third C–O bonds and there may also be some intermolecular effects such as restricted conformations and accessibility of catalytic surface sites. While the P–O dissociation reaction energy became less endothermic with increasing field strength, there was no detectable effect on the energy barrier. This suggests that the electric field stabilizes the products of the reaction but not the transition state.^114^

**Figure 12:**
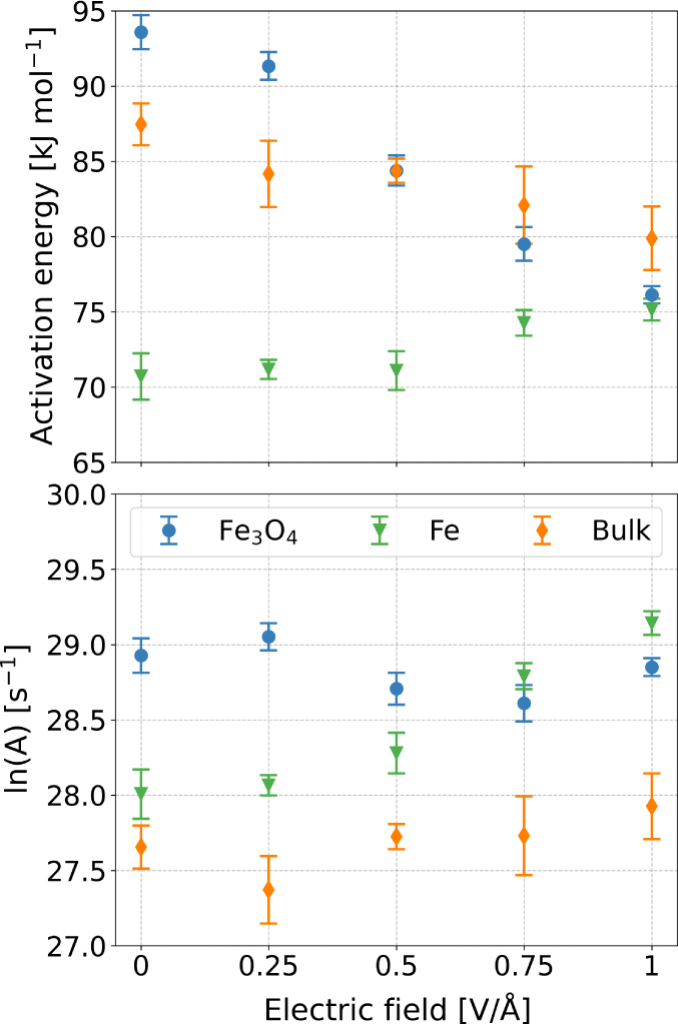
Dependence of the activation energy, *E*_a_, and pre-exponential factor, *A*, on the electric field strength for bulk TNBP, TNBP confined between Fe_3_O_4_ surfaces, and TNBP confined between nascent Fe surfaces.

Figure 12 shows the changes in *E*_a_ and ln(*A*) with electric field strength for the three studied systems. For all of the conditions studied, the reactivity of TNBP increases in the order: bulk < Fe_3_O_4_ < Fe. Without an external electric field, *E*_a_ is slightly increased for Fe_3_O_4_ relative to the bulk case but is significantly decreased for the Fe surface. On the other hand, ln(*A*) is similar for the bulk and Fe systems but is much higher for the Fe_3_O_4_ surface. External electric fields increase reactivity in our systems in two main ways: (i) directly reduce the activation energy by increased molecular polarization elongation of the C–O bonds and (ii) increase the number of successful collisions, which is manifested in eq 11 as an increase in *A*. For the bulk and Fe_3_O_4_ systems, the increase in reactivity under an applied electric field can be explained by the fact that the activation energy reduces approximately linearly with electric field strength, without any significant change in ln(*A*). At the strongest field strength (1 V/Å), the activation energy is similar for all three systems, suggesting that the reactivity is dominated by the electric field rather than collisions with the catalytic surfaces. Conversely, for the Fe system, the increase in reactivity is due to an increase in ln(*A*) and a slight increase in *E*_a_ is observed. This can be explained based on the very high reactivity of the catalytic Fe surface toward TNBP.^47^ This means that the increased reactivity under an applied electric field is due to increased collisions between the molecules and the surface rather than a direct reduction in activation energy, which is already very low for the Fe surfaces without an electric field. This is consistent with observations for other electrocatalytic processes,^112^ such as voltage-driven water dissociation on a TiO_2_–P25-nanoparticle catalyst.^111^

In this study, we have shown how external electric fields can influence both the activation energy and pre-exponential factor in the Arrhenius equation for the dissociation of TNBP, both in the bulk and when confined between ferrous surfaces. This study paves the way for future NEMD simulations capable of studying the combined effect of electric and mechanical energy on chemical reactivity, as is relevant to conditions inside tribological contacts with external electric fields^13^ as well as for electric field-assisted mechanochemical synthesis.^115^

## Conclusions

In this study, we have used NEMD simulations with ReaxFF to investigate the effect of electric fields on the adsorption and decomposition of phosphate esters in a bulk system and nanoconfined between ferrous surfaces. We first compared results obtained with two charge equilibration methods, the QEq method, and the QTPIE method. The QTPIE method demonstrates more accurate predictions of electric-field-induced polarizations and charge distribution within the TNBP molecules. A shortcoming of the QEq method is the long-range charge transfer strategy, which results in higher atomic charges compared with the QTPIE method. The inability to predict electric field-induced polarization using the QEq method limits its accurate descriptions of changes in kinetic energies and bond stretching under external electrostatic fields. Using the QTPIE method, the length of the C–O bonds increases under external electric fields due to the polarization. This study demonstrates that QTPIE is more suitable than QEq for the NEMD simulation of the thermal decomposition of molecules under external electric fields.

The rate of decomposition is higher in the nanoconfined system than that in the bulk system due to the catalytic action of the surfaces. When electric fields are applied to the confined systems, the phosphate anions are pulled toward the surface with a high electric potential, while the alkyl cations are pulled to the surface with a lower potential. This observation is consistent with previous tribology experiments, where asymmetric tribofilm growth has been reported, with polyphosphate tribofilms growing mostly on the surface with a high electric potential. Enhanced decomposition of the phosphate esters was observed in the presence of an external electric field. The main decomposition pathway, C–O bond cleavage, is enhanced under an electrostatic field as a result of the field-induced polarization. Reaction kinetic analysis through the Arrhenius equation for nascent iron and iron oxide surfaces suggests different contributors to the increase in reactivity under the electric fields. On the more reactive nascent iron surfaces, the increase in reactivity is contributed by the increased surface-molecule collisions under electric fields. While on the iron oxide surface, a higher reactivity under a stronger electric field is driven by the direct reduction in the activation energy for C–O bond breaking. This observation was verified in single-molecule NEB calculations, which show that the energy barrier is decreased upon application of an electric field through stabilization of the charged transition state. These findings provide important insights into the effect of electric fields on the thermal decomposition of nanoconfined molecules. The study also paves the way for future NEMD simulations to investigate the possibility of developing control strategies that utilize external electric fields to optimize tribofilm growth to protect engineering interfaces inside tribological contacts.

## Data Availability

All data reported in the paper can be obtained by emailing the corresponding author or tribology@imperial.ac.uk. The raw simulation data has also been deposited in a public Zenodo repository available at 10.5281/zenodo.12549989.

## Supporting Information Available

Additional figures and results for the dipole moment, system snapshots, the change in the number of bonds, reaction parameters from the Arrhenius equation, and the NEB calculations (PDF)
