# Supplementary material for: Effect of Electric Fields on the Decomposition of Phosphate Esters
Source: J Phys Chem C Nanomater Interfaces. 2024 Sep 12;128(38):15959–73. doi: 10.1021/acs.jpcc.4c04412 (PMC11440609; doi:10.1021/acs.jpcc.4c04412)
Supplement: Supplementary file 1 — jp4c04412_si_001.pdf [file jp4c04412_si_001.pdf]

# Supporting Information: Effect of Electric Fields on the Decomposition of Phosphate Esters

Zhaoran Zhu,[*,†] James P. Ewen,[*,†] Efstratios M. Kritikos,[‡,†] Andrea Giusti,[†] and Daniele Dini[†]

†*Department of Mechanical Engineering, Imperial College London, London SW7 2AZ, UK*

‡*Department of Applied Physics and Materials Science, California Institute of Technology, Pasadena, California 91125, USA*

E-mail: zhaoran.zhu22@imperial.ac.uk; j.ewen@imperial.ac.uk

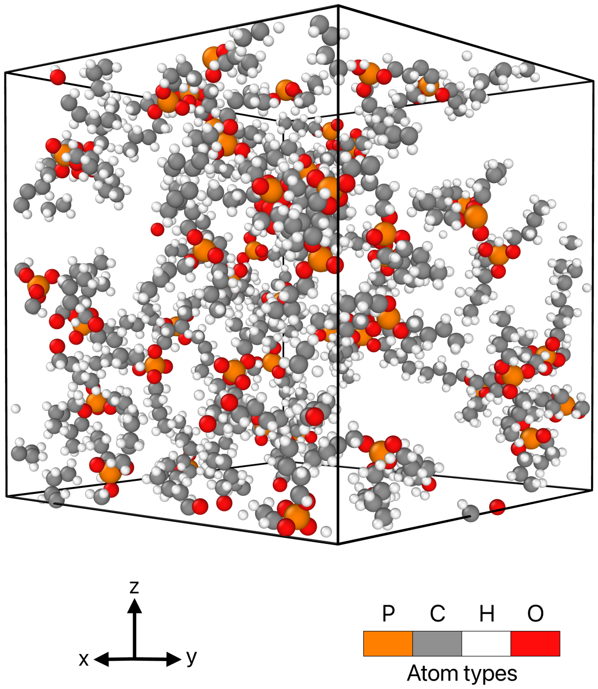


Figure S1: Snapshot for bulk systems containing 48 TNBP molecules.

## Effective electronegativity under electric fields

From Eq. 10,

$$\chi_{eff,i} = \frac{\sum_j (\chi_i^0 - \chi_j^0 + \vec{r_j} \cdot \vec{E}) S_{ij}}{\sum_{j'} S_{ij'}} - \vec{r_i} \cdot \vec{E}$$

Isolating the $\vec{E}$ term, got,

$$\begin{aligned}
\chi_{eff,i}|_{\vec{E}} &= \frac{\sum_j \vec{r_j} \cdot \vec{E}\, S_{ij}}{\sum_{j'} S_{ij'}} - \vec{r_i} \cdot \vec{E} \\
&= \sum_j \vec{r_j} \cdot \vec{E} \frac{S_{ij}}{\sum_{j'} S_{ij'}} - \vec{r_i} \cdot \vec{E} \text{ , where the distance decay function } f_{ij} = \frac{S_{ij}}{\sum_{j'} S_{ij'}} \\
&= \sum_j f_{ij}\, \vec{r_j} \cdot \vec{E} - \vec{r_i} \cdot \vec{E} \text{ , with } \vec{r_i} = \vec{r_i'} - \vec{r_0} \\
&= \sum_j f_{ij}\, (\vec{r_j'} - \vec{r_0}) \cdot \vec{E} - (\vec{r_i'} - \vec{r_0}) \cdot \vec{E} \\
&= \sum_j f_{ij}\, \vec{r_j'} \cdot \vec{E} - \sum_j f_{ij}\, \vec{r_0} \cdot \vec{E} - \vec{r_i'} \cdot \vec{E} + \vec{r_0} \cdot \vec{E}
\end{aligned}$$

where $\sum_j f_{ij} = 1$, the origin dependent term $\vec{r_0} \cdot \vec{E}$ is cancelled out. Finally, got,

$$\chi_{eff,i}|_{\vec{E}} = \sum_j f_{ij}\, \vec{r_j'} \cdot \vec{E} - \vec{r_i'} \cdot \vec{E} \tag{S1}$$

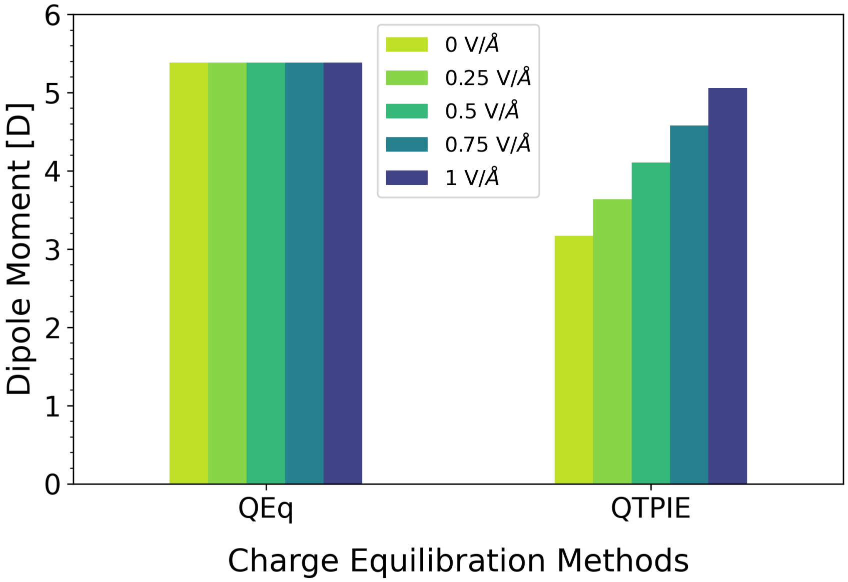


Figure S2: Dipole moment of an isolated TNBP molecule using (a) the QEq method and (b) the QTPIE method.

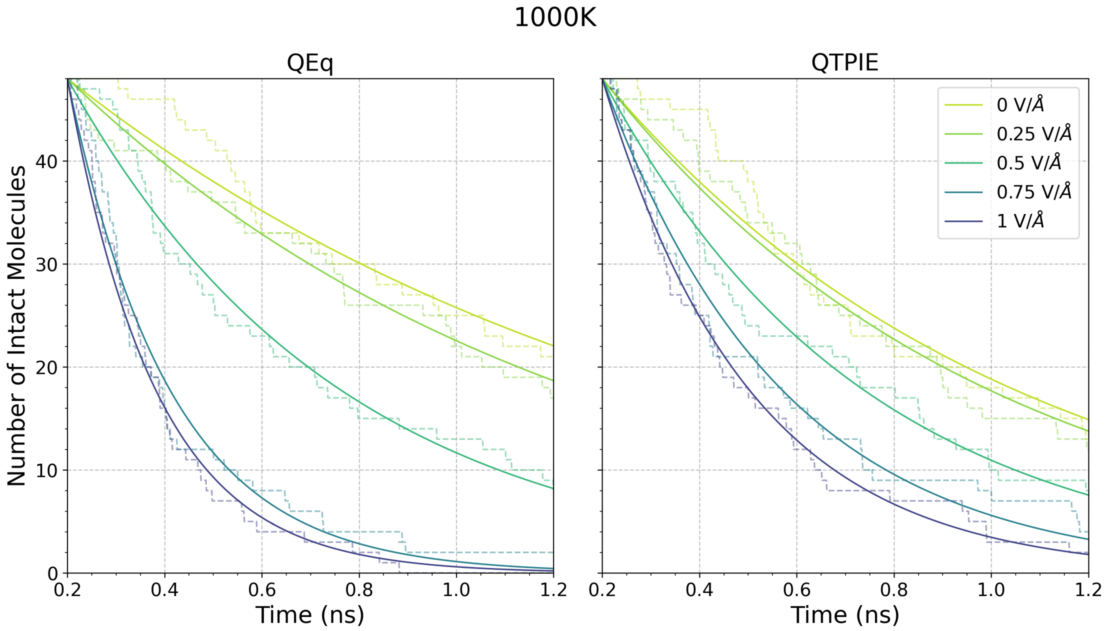


Figure S3: Change in the number of intact TNBP molecules confined between $Fe_3O_4$ surfaces at 1000 K under different electric field strengths using the QEq and the QTPIE method.

Table S1: Summary of activation energy $E_a$, and pre-exponential factor $A$ for TNBP with (a) bulk system, (b) $Fe_3O_4$ surface, and (c) nascent Fe surface.

(a) Bulk system

| **Electric Fields** | [V/Å] | 0 | 0.25 | 0.5 | 0.75 | 1 |
|---|---|---|---|---|---|---|
| $E_a$ | [kJ/mol] | 87.5 ± 1.4 | 84.2 ± 2.2 | 84.4 ± 0.8 | 82.1 ± 2.6 | 79.9 ± 2.1 |
| $ln(A)$ | [$s^{-1}$] | 27.7 ± 0.1 | 27.4 ± 0.2 | 27.7 ± 0.1 | 27.7 ± 0.3 | 27.9 ± 0.2 |

(b) $Fe_3O_4$ surface

| **Electric Fields** | [V/Å] | 0 | 0.25 | 0.5 | 0.75 | 1 |
|---|---|---|---|---|---|---|
| $E_a$ | [kJ/mol] | 93.6 ± 1.1 | 91.3 ± 0.9 | 84.4 ± 1 | 79.5 ± 1.1 | 76.1 ± 0.6 |
| $ln(A)$ | [$s^{-1}$] | 28.9 ± 0.1 | 29.1 ± 0.1 | 28.7 ± 0.1 | 28.6 ± 0.1 | 28.9 ± 0.1 |

(c) Nascent Fe surface

| **Electric Fields** | [V/Å] | 0 | 0.25 | 0.5 | 0.75 | 1 |
|---|---|---|---|---|---|---|
| $E_a$ | [kJ/mol] | 70.7 ± 1.5 | 71.2 ± 0.6 | 71.1 ± 1.3 | 74.3 ± 0.9 | 75.2 ± 0.7 |
| $ln(A)$ | [$s^{-1}$] | 28.0 ± 0.2 | 28.1 ± 0.1 | 28.3 ± 0.1 | 28.8 ± 0.1 | 29.1 ± 0.1 |

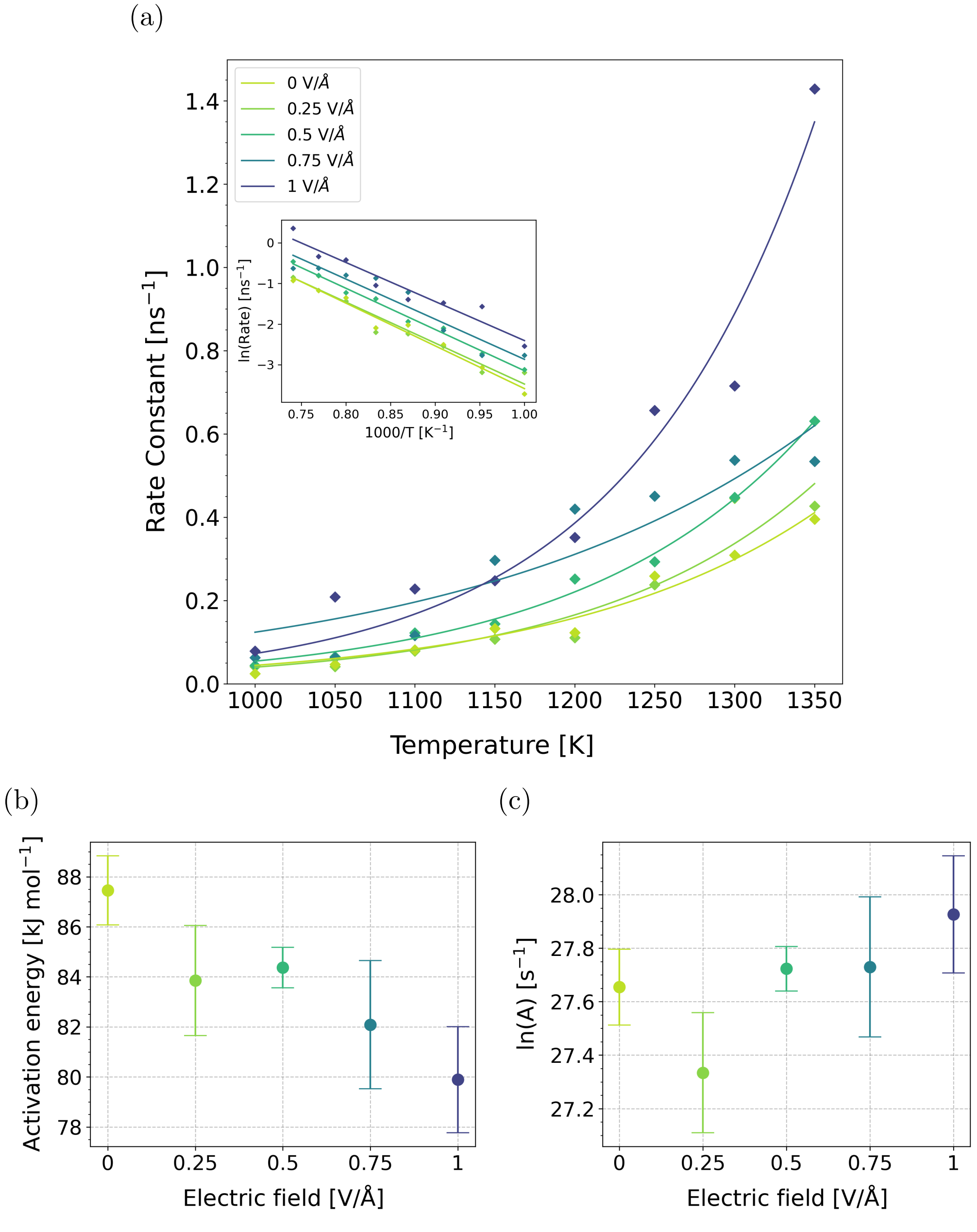


Figure S4: Effect of the electric fields on reaction kinetics for bulk TNBP system, (a) combined dependence from the electric field and temperature of C-O bonds scission rate; (b) change in activation energy, $E_a$ and (c) pre-exponential factor with external electric field strength.

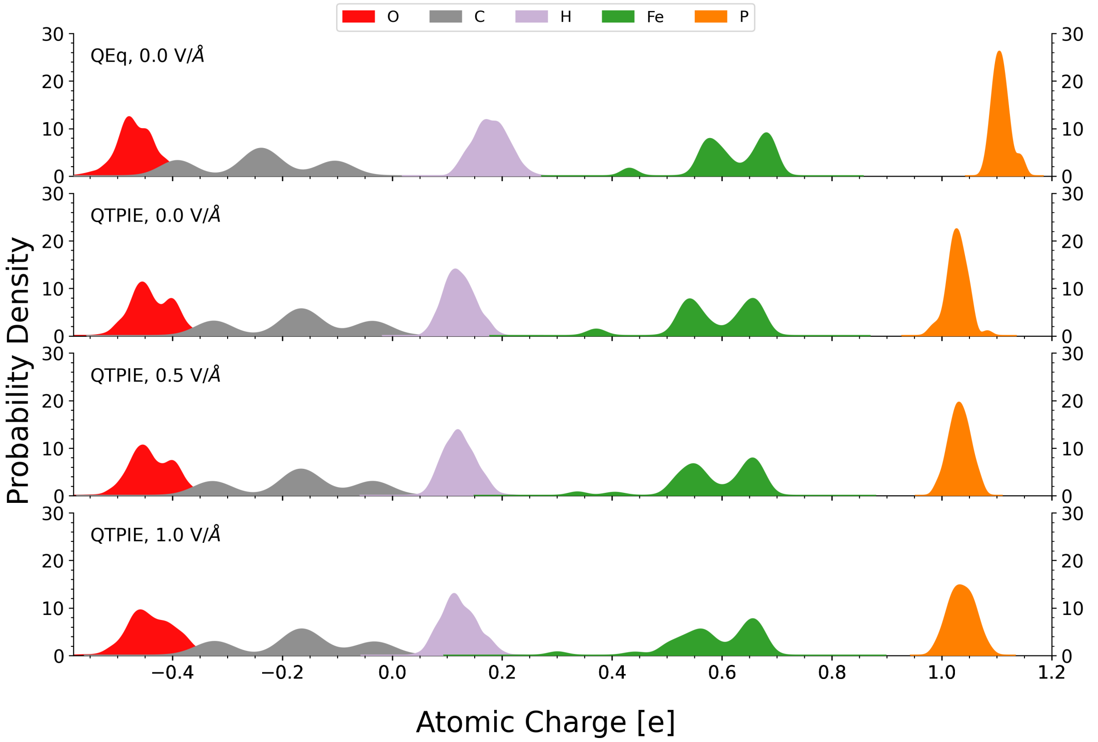


Figure S5: Atomic charges distribution of 48 TNBP molecules confined between $Fe_3O_4$ surfaces at 1100 K using the QEq method and the QTPIE method.

Table S2: Relative energies from NEB calculations using ReaxFF for (a) C–O cleavage; (b) P–O cleavage in a TNBP molecule on a $Fe_3O_4$ surface.

(a) C-O cleavage

| **Electric Fields** | [V/Å] | 0 | 0.25 | 0.5 | 0.75 | 1 |
|---|---|---|---|---|---|---|
| $\Delta E_{barrier}$ | [kJ/mol] | 138.7 | 125.3 | 118.6 | 108.1 | 95.4 |
| $\Delta E_{reaction}$ | [kJ/mol] | 110.3 | 72.2 | 33.0 | -10.4 | -57.3 |

(b) P-O cleavage

| **Electric Fields** | [V/Å] | 0 | 0.25 | 0.5 | 0.75 | 1 |
|---|---|---|---|---|---|---|
| $\Delta E_{barrier}$ | [kJ/mol] | 380.8 | 379.8 | 375.5 | 371.3 | 368.9 |
| $\Delta E_{reaction}$ | [kJ/mol] | 212.4 | 193.8 | 171.4 | 140.8 | 99.6 |

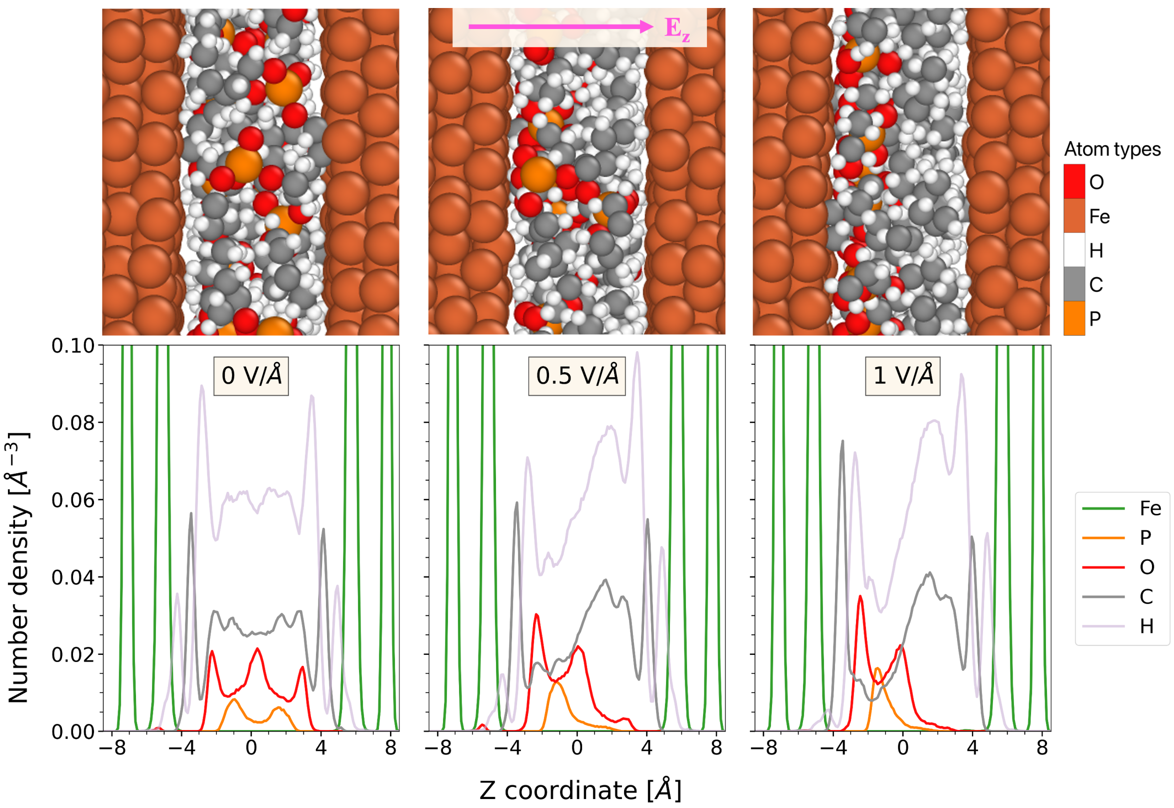


Figure S6: Simulation snapshots of 48 TNBP molecules confined between two $\alpha$-Fe surfaces at 1100 K under different electric field strengths (top), corresponding through-thickness number density profiles for different atom types (bottom). For both figures, the electric field was applied along the positive $z$ direction as shown by the pink arrow.

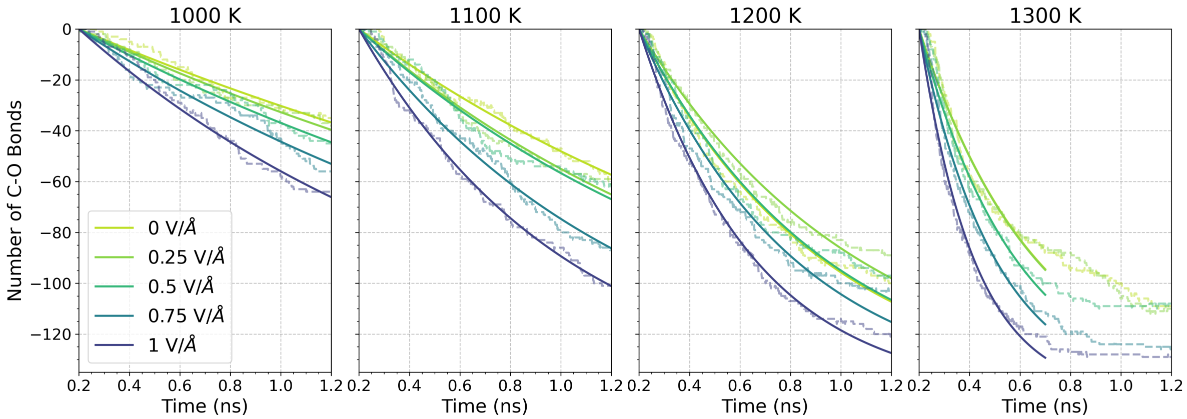


Figure S7: Change in the number of intact C-O bonds within TNBP molecules between nascent Fe surfaces under applied electric fields. Simulations were run with nascent $\alpha$-Fe (110) surfaces under 1200 K, and nascent $\gamma$-Fe (111) surfaces above 1200 K. The dashed line represents the decay in the number of C-O bonds from the MD simulations, while the solid line depicts the fitted curve of the decay using an exponential function. For simulations at 1300 K, the curve was exponential fitted with a shorter period of time (0.5 ns).

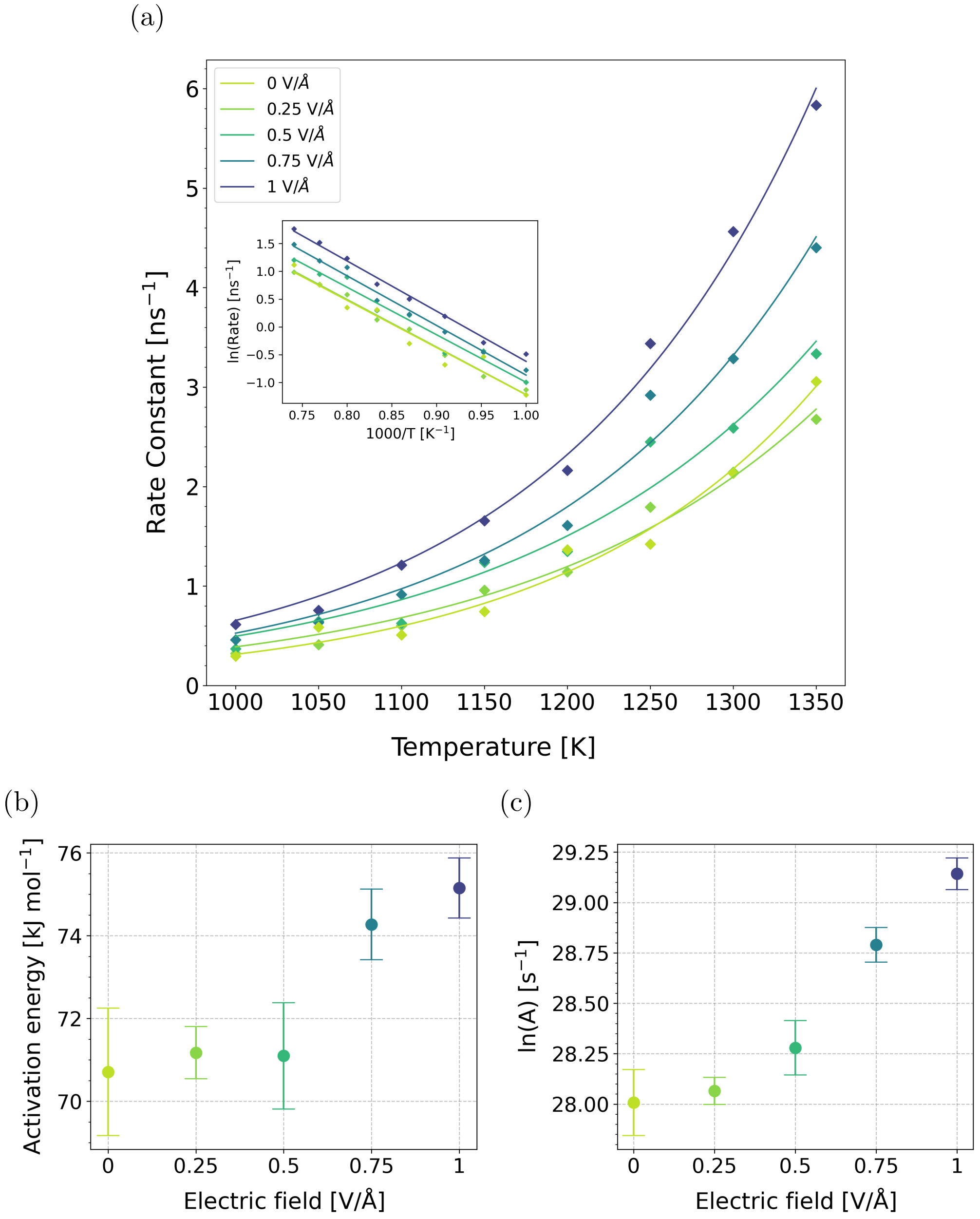


Figure S8: Effect of the electric fields on reaction kinetics for TNBP molecules confined between nascent Fe surfaces, (a) combined dependence from the electric field and temperature of C-O bonds scission rate; (b) change in activation energy, $E_a$ and (c) pre-exponential factor with external electric field strength.

(a) C-O cleavage

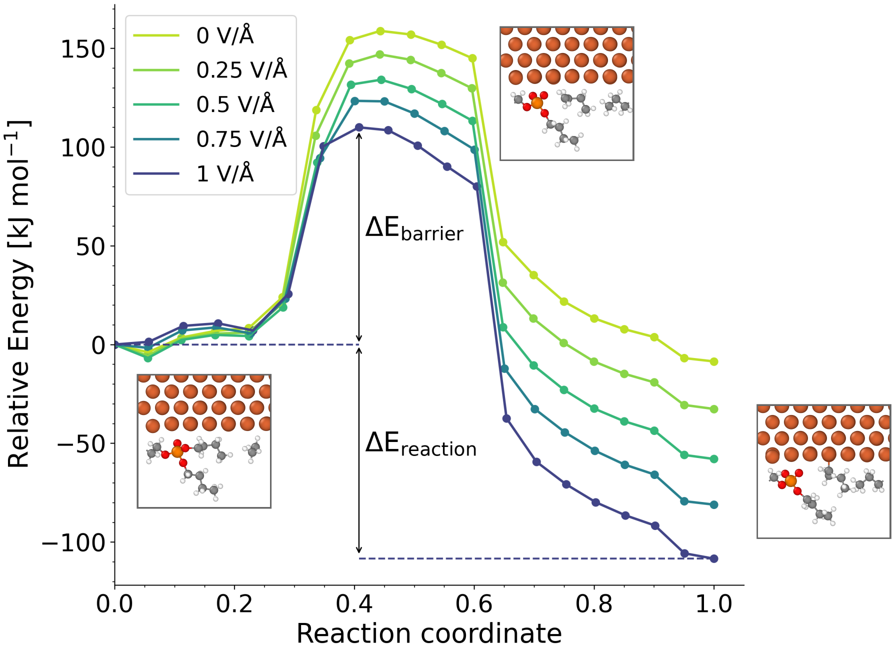


(b) P-O cleavage

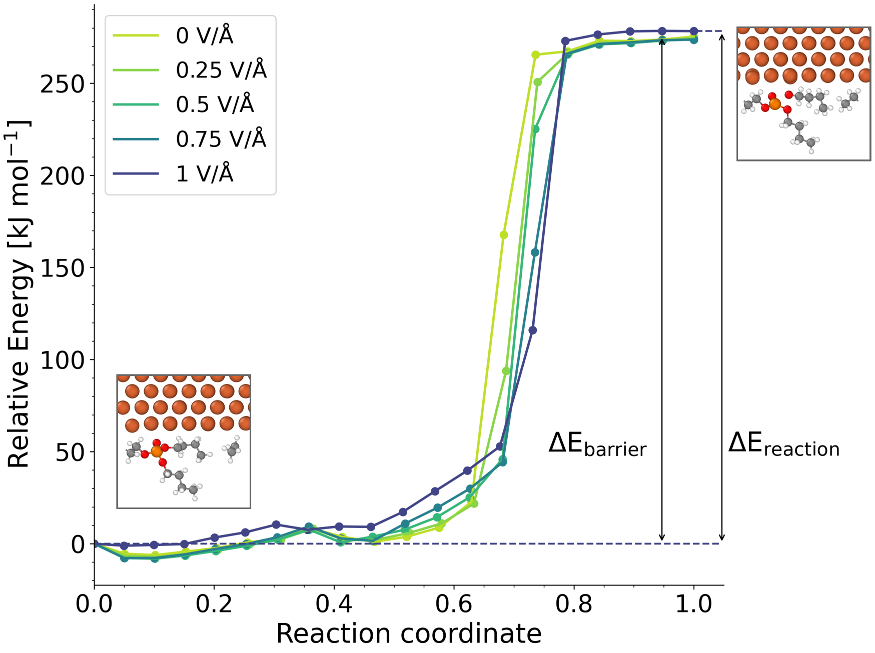


Figure S9: Relative energies and structures from NEB calculations for (a) C-O cleavage and (b) P-O cleavage in a TNBP molecule on an $\alpha$-Fe surface. The activation energy ($\Delta E_{barrier}$) and energy benefit ($\Delta E_{reaction}$) are illustrated based on the curve for the $E_z$ = 1 V/Åcase. The insert figures, from left to right, are the structures at the initial state, the state with the highest energy, and the final state.

Table S3: Relative energies from NEB calculations using ReaxFF for (a) C–O cleavage; (b) P–O cleavage in a TNBP molecule on an $\alpha$-Fe surface.

(a) C-O cleavage

| **Electric Fields** | [V/Å] | 0 | 0.25 | 0.5 | 0.75 | 1 |
|---|---|---|---|---|---|---|
| $\Delta E_{barrier}$ | [kJ/mol] | 158.8 | 146.9 | 134.1 | 123.4 | 109.9 |
| $\Delta E_{reaction}$ | [kJ/mol] | -8.6 | -32.6 | -57.9 | -81.1 | -108.4 |

(b) P-O cleavage

| **Electric Fields** | [V/Å] | 0 | 0.25 | 0.5 | 0.75 | 1 |
|---|---|---|---|---|---|---|
| $\Delta E_{barrier}$ / $\Delta E_{reaction}$ | [kJ/mol] | 275.4 | 274.5 | 273.9 | 273.7 | 278.4 |
